# The Efficacy and Safety of Carbon Ion Radiotherapy for Meningiomas: A Systematic Review and Meta-Analysis

**DOI:** 10.3389/fonc.2021.620534

**Published:** 2021-05-25

**Authors:** Jie-yun Li, Jing-wen Li, Yuan-chang Jin, Mei-xuan Li, Li-ping Guo, Zhi-tong Bing, Qiu-ning Zhang, Fei Bai, Xiao-hu Wang, Xiu-xia Li, Ke-hu Yang

**Affiliations:** ^1^ Evidence-Based Medicine Center, School of Basic Medical Sciences, Lanzhou University, Lanzhou, China; ^2^ Health Technology Assessment Center of Lanzhou University, School of Public Health, Lanzhou University, Lanzhou, China; ^3^ Key Laboratory of Evidence Based Medicine and Knowledge Translation of Gansu Province, Lanzhou, China; ^4^ The First School of Clinical Medicine, Lanzhou University, Lanzhou, China; ^5^ Institute of Modern Physics, Chinese Academy of Sciences, Beijing, China; ^6^ Lanzhou Heavy Ions Hospital, Lanzhou, China; ^7^ National Health Commission Medical Management Center, Beijing, China

**Keywords:** carbon ion radiotherapy, proton therapy, meningiomas, systematic review, meta-analysis

## Abstract

**Objective:**

The purpose of this systematic review and meta-analysis is to evaluate the efficacy and safety of carbon ion radiotherapy (CI-RT) in improving meningioma by comparing photon and protons radiotherapy.

**Methods:**

A comprehensive search for relevant studies published until March 17, 2021, was conducted in PubMed, the Cochrane Library, Chinese Biomedical Literature Database and EMBASE. Statistical analyses were performed with R 4.0.3.

**Results:**

We identified 396 studies, of which 18 studies involving 985 participants were included. Except for one low quality study, the quality of the included studies was found to be either moderate or high quality. The analyses conducted according random effects model indicated that the 1-year overall survival rate (OS) of benign and non-benign meningiomas after the CI-RT treatment was 99% (95%CL=.91-1.00, *I* ^2^ = 0%). The overall average 5-year OS for meningiomas was 72% (95%CL=0.52-0.86, *I* ^2^ = 35%), not as effective as proton radiotherapy (PR-RT) 85% (95%CL=.72-.93, *I* ^2^ = 73, Q=4.17, *df*=2, *p*=.12). Additionally, 5-year OS of atypical meningiomas (81%) was found to be significantly higher than anaplastic meningiomas (52%). The 10-year OS after CI-RT of patients with mixed grade meningioma was 91% (95%CL=.75-.97, *I* ^2^ = 73%). The 15-year OS after CI-RT 87% (95%CL=.11-1.00) or PR-RT 87% (95%CL=.23-.99, *I* ^2^ = 79%) were the same (Q=0, *df*=1, *p*=.99). After undergoing CI-RT for 3 and 5 years, the LC for benign meningioma was 100% and 88%, respectively, while the 2-year LC of non-benign meningiomas (atypical/anaplastic) was 33%. Headache, sensory impairment, cognitive impairment, and hearing impairment were found to be the most common adverse reactions, with individual incidences of 19.4%, 23.7%, 9.1%, and 9.1%, respectively.

**Conclusion:**

CI-RT is a rapidly developing technique that has been proven to be an effective treatment against meningioma. The efficacy and safety of CI-RT for meningiomas were similar to those of PR-RT, better than photon radiotherapy (PH-RT). However, there is a need for more prospective trials in the future that can help provide more supportive evidence.

## Introduction

Meningiomas are typically slow-growing, well-defined benign tumors that original from arachnoid cells. Meningioma is the most common primary non-glioma tumor in adults, and accounts for 25% of primary brain tumors (1). The annual incidence rate of meningiomas is approximately 8.3 in 100,000 (2). The World Health Organization (WHO) classifies meningiomas into three different categories, including grade I (benign), grade II (atypical) and grade III (malignant or anaplastic) (3). Although most meningiomas are benign, meningiomas do often adjoin or infiltrate key neurovascular structures. Furthermore, their growth can cause neurocognitive impairment and significant deterioration of their quality of life. Meningiomas are mostly diagnosed among middle-aged and elderly patients, but can also occur in young patients (4, 5). The frequency of meningiomas increases with age, and women are twice as likely to be diagnosed as men (6).

Benign meningiomas, including convex meningiomas and easily accessible skull base meningiomas, account for approximately 90% of all meningiomas (3, 7). Neurosurgical resection is considered to be the first choice of treatment for tumors that are easier to resect. Furthermore, there is no high risk of treatment-related side effects post-resection. In addition to surgery, a variety of radiation therapy (RT) methods are often used to strengthen local control of the tumor, particularly when surgery alone does not seem to be enough. Atypical and anaplastic meningiomas are characterized by more aggressive growth patterns, both of them are relatively rare tumors and only account for 4.7% and 2.8% of all meningiomas, respectively (8). Compared to patients with benign meningioma, they tend to have higher local recurrence and lower survival rate (9). Although traditional RT has been conducted across many meningioma treatments, it has been shown to significantly improve local control and prolong survival. However, the effect of treatment is still not satisfactory, and most patients tend to have recurrence during follow-up. In 1997, the Department of Radiation Oncology at the University of Heidelberg Hospital provided carbon ion therapy in Gesellschaft für Schwerionenforschung (GSI), Darmstadt, Germany. Carbon ion therapy involves the use of active beam transmission through raster scanning technology to irradiated patients with different brain and skull base tumors (10–12). The study demonstrated that all patients had good tolerance to carbon ion therapy. The 1-year local control rate was found to be 94%, and no severe toxicity or local recurrence within the treatment volume was observed. The clinical effect and technical feasibility of the carbocation therapy were announced. Carbon ion radiotherapy (CI-RT) is characterized through its unique physical and biological properties that allow for a gradual increase of dose deposition through a steep gradient. As a result, high-dose local therapy can be applied, while normal structures are likely to survive. Furthermore, tumors near normal dangerous organs may be treated more effectively with higher doses (13, 14). Additionally, CI-RT has a higher local tumor control rate, as well as increasing relative biological effectiveness (RBE), which is defined as the ratio of ion dose to photon radiotherapy (PH-RT). RBE fluctuates with environmental factors (15). The increasing RBE offers further potential radiobiological advantages, such as reduced repair capacity, decreased cell-cycle dependence, and possibly, stronger immunological responses (16).

Application of CI-RT for treating meningiomas is a currently developing research field. In recent years, clinical trials of CI-RT for meningiomas have gradually increased and have been able to evaluate overall survival rate (OS), local control rate (LC), tumor volume and additional indicators of meningioma after CI-RT. Hence, a meta-analysis for this small but heterogeneous body of evidence is needed and may be useful for further advancing the application and knowledge within this field. The present systematic review and meta-analysis analyzed all available literature for evidence of efficacy and safety of CI-RT for the treatment of meningiomas by comparing PR-RT and PH-RT.

## Method

The Cochrane Handbook for Systematic Reviews of Interventions and PRISMA Statement were used to guide the conduct and reporting of this review. A study search was done using four electronic databases, including PubMed, the Cochrane Library, Chinese Biomedical Study Database, and EMBASE (17). As a systematic review and meta-analysis, our study does not have ethical issues and therefore no need approval from institutional review board.

### Inclusion and Exclusion Criteria

Studies were included if they matched the following criteria: (a) patients with meningioma had been diagnosed by histopathology (b) the clinical treatments were carbon-ion, photon, or protons radiotherapy; (c) reported data that can be used to calculate the effectiveness and/or adverse effects; (d) prospective or retrospective clinical trials.

Publications were excluded if they were (a) case reports; (b) letters, editorials, protocols, reviews; (c) duplicate publications; (d) cell and animal experimental studies; (e) lacking detailed data.

### Data *S*ources and Search Strategy

A comprehensive search was conducted for relevant studies that were published in English or Chinese, databases including PubMed, the Cochrane Library, Chinese Biomedical Literature Database, and EMBASE on March 17, 2021. The search keywords including (“meningioma” OR “Meningiomas” OR “meningioma” OR “meningothelioma”) AND (“ion” OR “proton” OR “photon”). Details on the search strategy have been provided in **Supplementary Material** (18). The reference lists of the studies were searched manually to identify additional studies (19). The Clinical Trials.gov website was also searched for studies that were registered as completed but not yet published.

### Selection *C*riteria and Data Extraction

**Figure 1:**
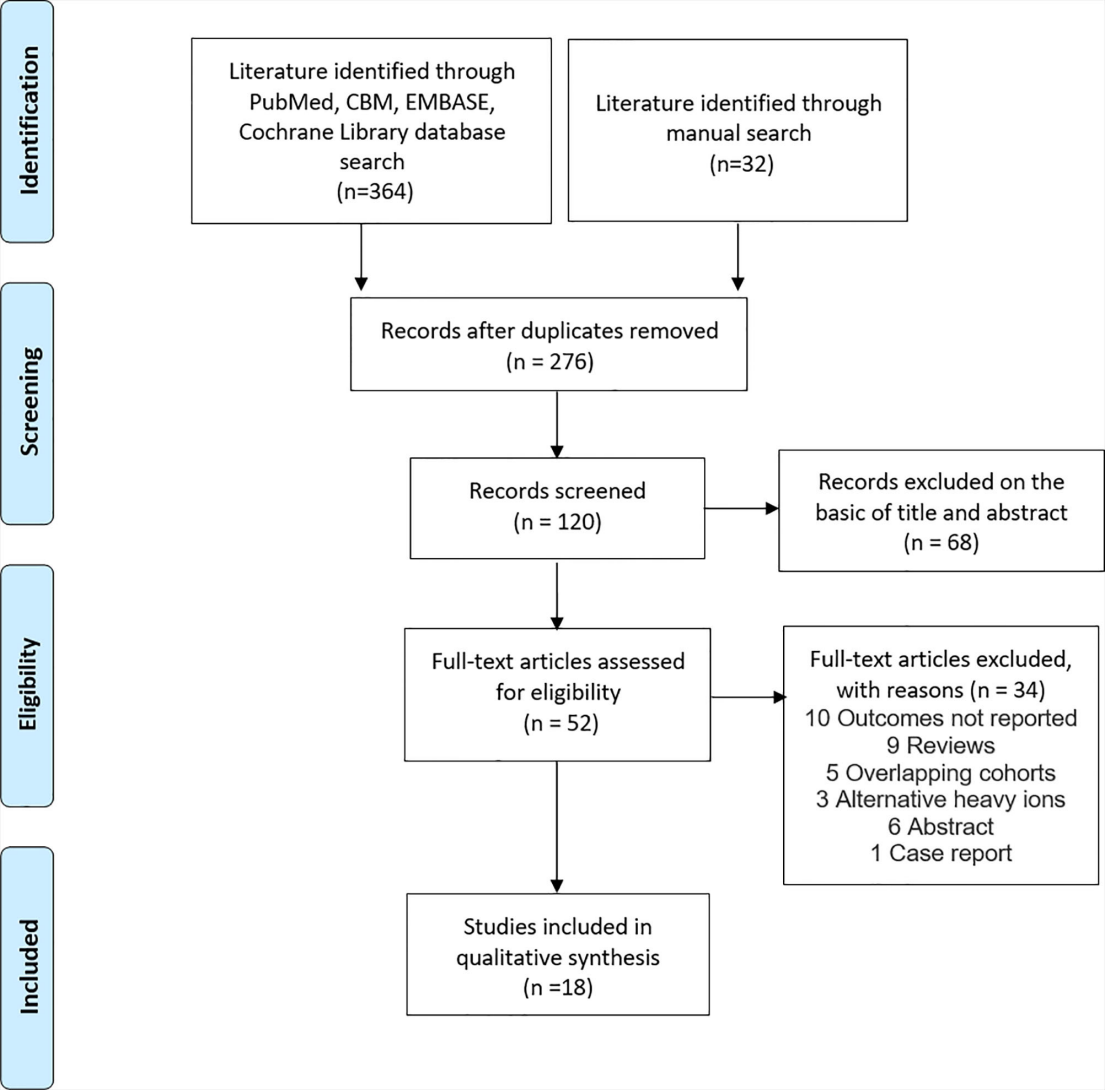
PRISMA flowchart showing study selection.

The titles and abstracts of studies identified in the databases were screened by two reviewers (J-WL and J-YL) independently with a standardized approach. We retrieved the full-text articles of all potentially eligible studies (20). We resolved any disagreements about research qualifications by discussing or consulting the third reviewer (Y-CJ or M-XL). A flow diagram of the systematic search and study selection process is shown in **Figure 1**.

Eligible studies were screened in their entirety and developed a data extraction form, and the information including authors and year of publication, publication type, type of treatment, sample size, WHO grade, total dose, duration of intervention and follow-up were recorded in an Excel spreadsheet. We pre-tested it on five studies and subsequently adapted the final version. If the inclusion criteria were met, the full-text of each study was coded by the first authors (J-YL and J-WL) using this template.

If there was any evidence for the use of the same sample in different publications, authors were contacted for clarification (21). If it was confirmed that two studies were based on the same data, we chose the study that reported the most comprehensive results (17). If a study conducted multiple interventions and targeted with different population, each intervention was considered as an independent report.

### Heterogeneity, *S*ensitivity, and Publication Bias

Based on the Cochrane Handbook Version 6.1.0, 2020, heterogeneity was assessed using Q-test to estimate the standard deviation of the true effect sizes (22). A significant Q-test indicates that effect sizes of primary studies do not belong to the same distribution of effect sizes. When performance qualification statistics *p*≥.05, was considered no significant heterogeneity among the included studies (21). *I* ^2^ index is used to the estimated amount of variability in the true effect sizes, and the proportion of observed variability that can be explained by true heterogeneity. 25%, 50%, and 75% of *I^2^* index indicate low, moderate, and high degrees of heterogeneity, respectively.

“Leave-one-out” method is used in sensitivity analyses to check for outliers that potentially influence the results of the meta-analysis disproportionately (21). All analyses were performed repeatedly with each study removed once to detect whether overall results effect on a single study.

Publication bias means that statistically significant results are more likely to be published, while statistically insignificant results are less likely to be published. Therefore, these studies with no significant significance could be more likely to remain in the “file drawer” (23). Publication bias was assessed by three methods. Funnel plots illustrate the effect sizes of primary studies as a function of study precision. Asymmetry in plots can indicate publication bias (24). Egger’s regression test yields a statistical verification of funnel plot asymmetry. If any bias could be assumed based on these analyses, we planned to apply the trim-and-fill procedure to estimate the unbiased overall effect (25).

### Risk of Bias Assessment

In order to assess the quality of the case series, the authors (J-WL and J-YL) independently assessed bias using an evaluation scale that was developed by the Canadian Institute of Health Economics (IHE) (26). The authors evaluated the biases to create their own research list that meets the inclusion and exclusion criteria. The third author (Y-CJ) examined differences between the two lists. The difference was resolved through discussion between the three authors. The IHE case series methodology quality evaluation list is composed of a total of 20 setting items. Each of the items were assessed as “yes,” “no,” and “unclear.” Trials that had more than 14 “yes” components were identified as having a moderate risk of bias (**Supplementary Table 1**).

### Data Synthesis and Statistical Analysis

The fixed-effects model and the random-effects model are based on different assumptions. The results of meta-analysis using fixed-effect models are limited to specific populations (27). As the fact that the studies were conducted under different conditions (e.g., cancer grade, intervention, etc.) could indispensably cause differences among the results. Thus, in the synthesis of effect sizes during the present meta-analytical processes, analyses were conducted according to the random effects model. We computed proportion with 95% confidence intervals (95%CI) to estimate effect sizes for continuous outcomes. Besides, we use stratified analysis to explore subgroup analysis. The whole process of data analysis was performed in R 4.0.3. with the ‘meta’ package.

## Result

### Selection and Characteristics of Studies

Among the 396 studies that were related to ion, proton or photon radiotherapy were identified, 52 were selected for full-text review. Eventually, 18 studies were included in the meta-analysis (see **Figure 1**) **(** 28–42). 12 studies reported OS (28–31, 35, 36, 38, 41–43), 17 studies reported LC rates (29–43), and nine studies reported toxic reactions (29, 31, 32, 34, 35, 38, 39, 41, 42). Eight studies about CI-RT were all from Heidelberg, Germany and were published between 2010 and 2018 (28, 30, 31, 36, 43–45). Among these studies, two prospective studies (28, 43), while the remaining six were retrospective studies (30, 31, 38, 44, 45). The number of patients that were included in each study ranged from 8 to 110, the follow-up ranged from 2 to 243 months. Eight studies about PR-RT were from four various countries, three of them from Switzerland (35, 41, 42), two from Sweden (32, 40), two from the United States (33, 37) and South Africa (39). The number of patients that were included in each study ranged from 13 to 170, the follow-up ranged from 32 to 207 months. Three studies were on photons or photons combined with protons. Two of them were from the United States (34, 36) and one from France (29). The number of patients included in each study ranged from 24 to 44, the follow-up ranged from 1 to 193 months. CI-RT was applied at a median dose of 18 Gy E, while PH-RT was applied at a dose of 50 to 50.4 Gy E and PR-RT was applied with a dose of 21.9 to 57.6 Gy E. In these studies, at least 433 patients were with benign meningioma (WHO grade I), while 138 patients were with atypical meningioma (WHO grade II), and 32 patients were with anaplastic meningioma (WHO grade III). Characteristics of these studies are shown in **Table 1**.

**Table 1:** Characteristics of the study.

Study	Institute	Design	Patient(n)	Age(Median)	WHO GRADE(N)				Intervention	Radiation modality	Follow up (Month)	Overall survival	Local control	Toxicity Note
					I	II	III	Unknow						
**Gudjonsson et al.** (**32**)	**Uppsala. Sweden**	**Retrospective cohort**	**19**	52 (34–66)	**15**	**/**	**/**	**4**	**Proton**	**24 Gy, 6 Gy fr**	**40 (12**–**115).**	**/**	**100% at 3-year**	**No toxicity. No additional cranial nerve dysfunctions have occurred during follow-up.**
**Hug et al.** (**34**)	**MGH, Boston MA.USA**	**Retrospective cohort**	**31**	**60 (33**–**85)**	**/**	**15**	**16**	**/**	**Photo or** **Proton+ Photon**	**62.5 (50.4-68.4)Gy/CGE**	**48**	**/**	**50% at 5 years for atypical, 19% at 8 years for malignant**	**One patient developed radiation necrosis. The investigators did** **not report when this occurred relative to treatment**
**Vernimmen et al.** (**39**)	**Tygerberg, South Africa**	**Retrospective cohort**	**23**	**45.6 (7.2**–**64.8)**	**23**	**/**	**/**	**/**	**Proton**	**54 Gy in 27 fr to 61.6 Gy in 16 fr**	**40(13**–**69)**	**/**	**88% at 5 years**	**1 patient developed short-term memory disturbance**
**Weber et al.** (**41**)	**PSI, Switzerland**	**Retrospective cohort**	**13**	**/**	**11**	**2**	**/**	**/**	**Proton**	**56 Gy (52.2–64 Gy)**	**34.1**	**84.6% at 3 years**	**100% at 3 years**	**Cumulative 3-year toxicity free survival**
**Boskos et al.** (**29**)	**CPO, Orsay, France**	**Retrospective cohort**	**24**		**/**	**19**	**5**	**/**	**Photo or** **Proton+ Photon**	**68(56–68)Gy/CGE**	**48(1–87)**	**100% at 1-year** **95.5% at 2-year** **80.4% at 3-year** **65.3% at 4-year** **53.2% at 5-year** **46.6% at 8-year**	**82.9% at 1-year** **82.9% at 2-year** **61.3% at 3-year** **61.3% at 4-year** **46.7% at 5-year** **47.7% at 8-year**	**Most common possible complications induced** **are neuropathy, radiation necrosis, and insufficiency of the** **pituitary gland.**
**Combs 2010a** (**46**)	**Heidelberg Germany**	**Retrospective cohort**	**8**	**52**	**/**	**/**	**/**	**8**	**Carbon lon Boost, Photons**	**Carbon ion, 18 Gy E; Photon, 50.4 Gy E**	**77(6**–**108)**	**75% at 5 years**	**86% at 5 years** **72% at 7 years**	**/**
**Halasz et al.** (**33**)	**MGH, Boston MA.USA**	**Retrospective cohort**	**50**	**60 (33–85)**	**50**	**/**	**/**	**/**	**Proton**	**13 Gy (10–15.5) in 1 fr**	**32(6**–**133)**	**/**	**94% at 3 years**	**/**
**Adeberg et al.** (**28**)	**Heidelberg Germany**	**Prospective cohort**	**85**	**55**	**/**	**85**		**/**	**Proton and carbon ion boost**	**57.6 Gy E**	**73 (3**–**243)**	**81% and 53% at 5 years for atypical or anaplastic**	**/**	**/**
**Rieken et al.** (**38**)	**Heidelberg Germany**	**Retrospective cohort**	**7**	**42(7**–**77)**	**3**	**3**	**1**	**/**	**Proton and carbon ion boost**	**Carbon ion, 18 Gy E; Proton, 52.2–57.6 Gy E.**	**4.5**	**100%**	**100%**	**/**
**Slater et al.** (**37**)	**Loma Linda University Medical Center, USA**	**Retrospective cohort**	**47**	**54.2 (22–85)**	**/**	**/**	**/**	**47**	**Proton**	**59 Gy**	**74**	**/**	**99% at 5 years**	**13% of patients developed neurologic symptoms 6% of patient presented pan hypopituitarism**
**Weber et al.** (**42**)	**PSI, Switzerland**	**Retrospective cohort**	**39**	**48.3**	**/**	**/**	**/**	**39**	**Proton**	**56(52.2–64 Gy)**	**62**	**82% at 5 years**	**84.6% at 5 years**	**Cumulative 5-year Grade 3 late toxicity-free survival**
**Combs 2013a** (**43**)	**Heidelberg Germany**	**Retrospective cohort**	**107**	**48(1**–**85)**	**/**	**/**	**/**	**107**	**Proton and carbon ion boost**	**Carbon ion, 18 Gy E; Proton, 52.2 – 57.6 GyE.**	**12(2**–**39)**	**100% at 3 years**	**54% at 1 year** **33% at 2 years**	**/**
**Combs 2013b** (**41**)	**Heidelberg Germany**	**Prospective cohort**	**70**	**55(27**–**83)**	**30**	**23**	**4**	**13**	**Carbon lon Boost, Photons**	**Photon, 50 Gy E; carbon ion, 18 Gy E.**	**6(2**–**22)**	**100% at 1 years**	**100% at 1 years**	**/**
**Murray et al.** (**35**)	**PSI, Switzerland**	**Retrospective cohort**	**96**	**/**	**61**	**35**		**/**	**Proton**	**54(50.4–64 Gy)**	**56.9 (12.1-207.2)**	**92% and 80% at 5 years for benign or non-benign**	**95% and 69% at 5 years for benign or non-benign**	**5 years grade III Free survival=89.1%**
**Sanford et al.** (**36**)	**MGH, Boston MA.USA**	**Retrospective cohort**	**44**	**9-87**	**/**	**44**	**/**	**/**	**Proton+ Photon**	**55.8 Gy or 63.0 Gy**	**73 (3**–**193)**	**100% and 92% at 15 years for high and low dose**	**99% at 10 year** **91% at 15 years**	**/**
**Vlachogiannis et al.** (**40**)	**Uppsala. Sweden**	**Retrospective cohort**	**170**	**54.2 (22–85)**	**170**	**/**	**/**	**/**	**Proton**	**21.9 (14–46 Gy)**	**84**	****	**93% at 5 year** **85% at 10 years**	**/**
**El Shafie 2018a** (**30**)	**Heidelberg Germany**	**Retrospective cohort**	**110**	**53**	**60**	**7**	**1**	**42**	**Proton and carbon ion boost**	**Proton, 54 Gy E; Carbon ion 18Gy E.**	**46.8(34.3**–**61.7)**	**96.2% at 5 years** **92% at 6 years**	**100% at 3years** **96.6% at 5 years**	**/**
**El Shafie 2018b** (**31**)	**Heidelberg Germany**	**Retrospective cohort**	**42**	**54**	**10**	**25**	**6**	**1**	**Proton and carbon ion boost**	**Proton, 54 Gy E; Carbon ion 19Gy E.**	**49.7**	**89.6% at 1-year** **71.4% at 2 years**	**71% at 1 year** **56.5% at 2 years**	**No grade 4 or 5 toxicities were observed**

### Risk of Bias

As shown in the **Supplemental Table 1**. Except for one low quality study (32), the quality of the included studies was found to be either moderate or high quality. All the assumptions, and objectives of included studies were described in detail, as well as the characteristics of the patients and interventions. All included studies used reasonable methods and statistical tests to measure relevant outcome indicators. Meanwhile, reported the duration of follow-up and the number of people lost to follow-up and the reasons. But it’s worth noting that only 16 percent of all studies were multicenter. Inclusion and exclusion criteria were elaborated in 56.5% of the studies. Whether the inclusion of patients was continuous is unknown in 50% of the studies. 38.9% of the joint intervention measures were clearly described. 11.1% of the studies were prospective studies, and it was unclear of all included studies that whether or not to blind the outcome evaluator.

### Overall *S*urvival *R*ate

**Figure 2:**
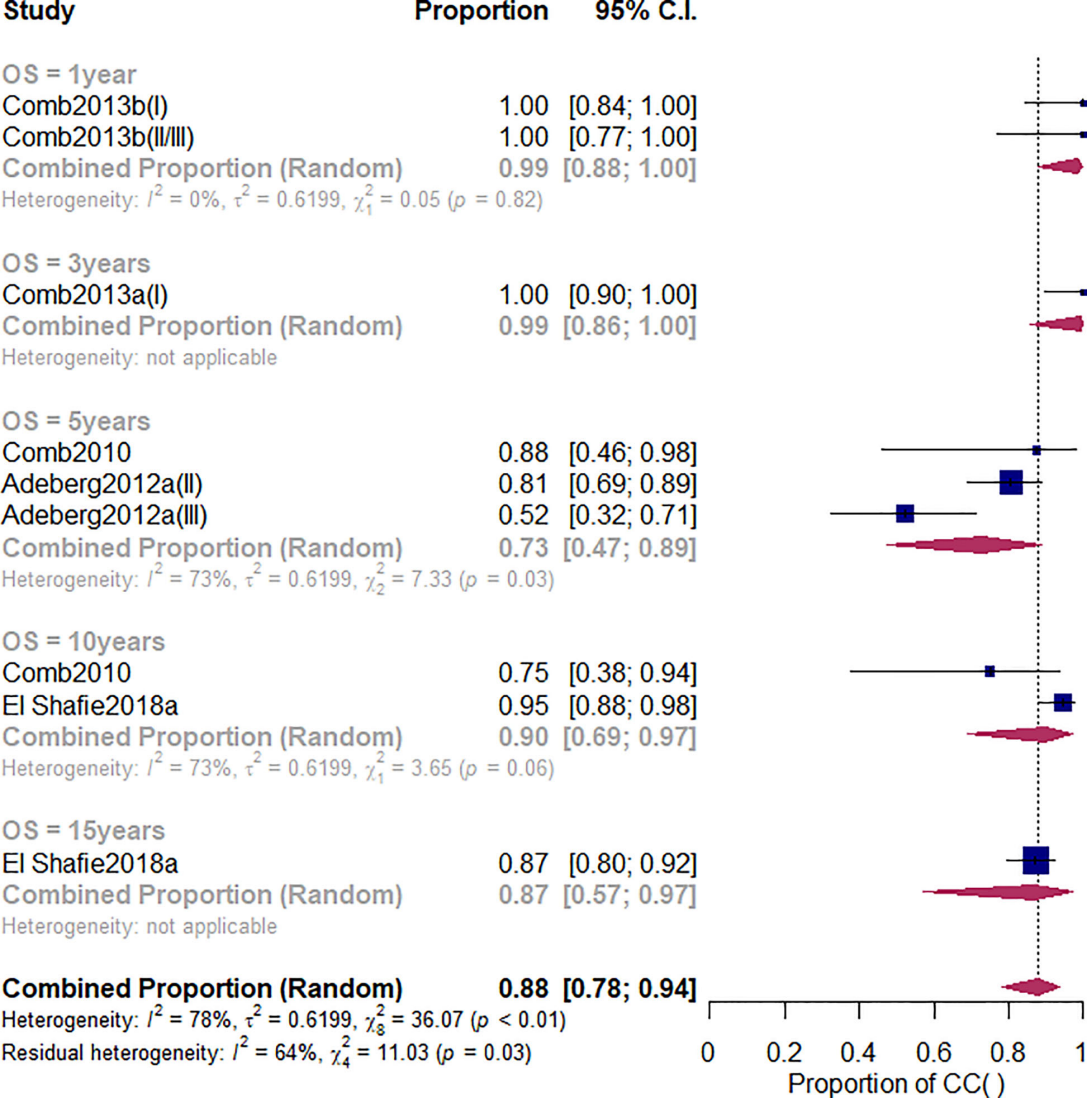
The overall survival rate of meningioma initial diagnosis treated with CI-RT.

Overall survival (OS) is one of the study’s primary outcomes. The duration of survival is the time interval between an initial diagnosis (date of the neuropathology report) and date of death due to any cause. Patients that were not reported to be dead or lost to follow-up were censored at the date of the last follow-up examination. Among the studies related to the CI-RT, five studies (28, 30, 44, 45) reported the OS of patients with meningioma (**Figure 2**). The 1-year OS, no matter benign or non-benign meningiomas was 99% (95%CL=.91-1.00, *I* ^2^ = 0%). As shown in **Supplementary Figure 1** and **Table 2**, there was a significant difference among the three different treatments (Q=5.81, *df*=2, *p*=.04). The 3-year OS of CI-RT was 100% (95%CL=0.90-1) is better than that of PR-RT 85% (95%CL=0.55-0.96) and proton combined with photon radiotherapy 79% (95%CL=.59-.91). The overall average 5-year OS for meningiomas was 72% (95%CL=.52-.86, *I* ^2^ = 35%), not as effective as proton radiotherapy (PR-RT) 85% (95%CL=.72-.93, *I* ^2^ = 73, Q=4.17, *df*=2, *p*=.12) (**Supplementary Figure 2**). Additionally, 5-year OS of atypical meningiomas (81%) was found to be significantly higher than anaplastic meningiomas (52%). The 10-year OS after CI-RT of patients with mixed grade meningioma was 91% (95%CL=.75-0.97, *I* ^2^ = 73%). The 15-year OS after CI-RT 87% (95%CL=.11-1.00) or PR-RT 87% (95%CL=.23-.99, *I* ^2^ = 79%) were the same (Q=0, *df*=1, *p*=.99) (**Supplementary Figure 3**). The 1-year and 2-year survival rates of patients with recurrent intracranial meningiomas were 90% and 71%, respectively.

**Table 2:** Outcomes with various treatment methods for meningioma.

	Intervention	K	Proportion	95%CI	*I* ^2^	Q	*df*	*p*
**1-year OS**	Proton+photon	1	98%	0.75–1	-	0.04	1	0.84
	Carbon+photon	2	99%	0.91–1	0%			
****	Total	3	98%	0.92–1		****	****	****
**3-year OS**	Proton+photon	1	79%	0.59–0.91	-	5.81	2	<.05
	Proton	1	85%	0.55–0.96	-			
	Carbon+photon	1	100%	0.90–1	-			
****	Total	3	90%	0.65–0.98		****	****	****
**5-year OS**	Proton+photon	1	54%	0.23–0.83	-	4.17	2	0.12
	Proton	3	85%	0.72–0.93	73%			
	Carbon+proton	3	72%	0.52–0.86	35%			
****	Total	7	77%	0.66–0.85		****	****	****
**10-year OS**	Carbon+photon	2	91%	0.75–0.97	73%	-	-	-
**15-year OS**	Proton+photon	2	87%	0.23–0.99	79%	0	1	0.99
	Carbon+proton	1	87%	0.11–1	-			
****	Total	3	87%	0.36–0.99		****	****	****
**1-year LC**	Proton+photon	1	83%	0.63–0.94	-	5.46	1	<.01
	Carbon+photon	1	53%	0.37–0.68	-			
****	Total	2	69%	0.34–0.91		****	****	****
**2-year LC**	Proton+photon	1	83%	0.63–0.94	-	12.48	1	<.01
	Carbon+photon	1	33%	0.20–0.50	-			
****	Total	2	60%	0.14–0.94		****	****	****
**3-year LC**	Proton+photon	1	62%	0.42–0.97		17.28	2	<.01
	Proton	2	94%	0.85–0.98	0%			
	Carbon+photon	1	99%	0.9–1				
****	Total	4	83%	0.72–1		****	****	****
**5-year LC**	Proton+photon	3	41%	0.21–0.65	0%	16.46	3	<.01
	Proton	7	89%	0.80–0.95	72%			
	Carbon+proton	1	88%	0.36–0.99	-			
****	Total	11	79%	0.68–0.87		****	****	****
**8-year LC**	Proton+photon	3	29%	0.14–0.51	53%	-	-	-
**10-year LC**	Proton+photon	1	98%	0.86–1	-	3.71	1	<.05
	Proton	1	85%	0.79–0.90	-			
****	Total	2	93%	0.65–0.99				

### Sensitivity and Publication Bias

According to the “leave-one-out” strategy, the effect sizes estimated values of eight studies related to CI-RT from .74 to .87, indicated that there were no particularly prominent sensitivity issues in the included literature (**Supplementary Figure 4**). The shape of the funnel plots appeared symmetrical in the comparison model showed that most effect sizes seem to locate symmetrically upwards the graph, and scatter around both sides of the line. Egger’s regression test did not show a publication bias (*p*=0.26). Besides, there was no obvious change in the results after the trim and -fill estimate (**Supplementary Figure 5**). Besides, as shown in **Supplementary Figure 6**, different study designs were not the potential source of heterogeneity.

### Local Control Rate

**Figure 3:**
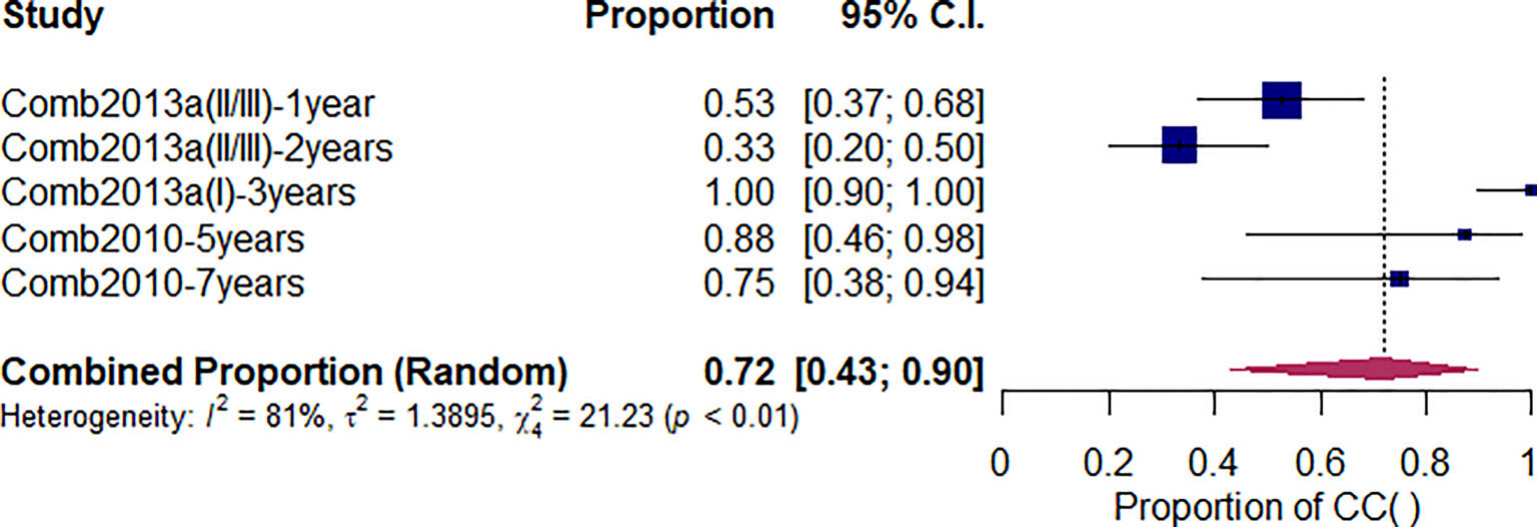
The local control rate of meningioma initial diagnosis treated with CI-RT.

As shown in **Figure 3**, after undergoing CI-RT for 3 and 5 years, the local control rate (LC) for benign meningioma was 100% and 88%, respectively, while the 2-year LC of non-benign meningiomas (atypical/anaplastic) was 33%. Compared with other treatments, the 3-year LC of CI-RT is better than PR-RT and proton combined with photon (99% *vs.* 94% *vs.* 62%, *Q=17.28, df=2, p<.01*) (**Supplementary Figure 5**). As shown in **Supplementary Figure 8** and **Table 2**, the 5-year LC of CI-RT combined with PR-RT was 88%(95%CL=.36-.99) same as the PR-RT 89% (95%CL=.80-.95, *I* ^2^ = 72), but significantly higher than PR-RT combined with PH-RT. 41% (95%CL=.21-.65, *I* ^2^ = 0, Q=16.46, *df*=3, *p*<.01)

### Toxic and Side Effect

According to the General terminology Standard for adverse events (CTCAEv4.0), grade I and II side effects are classified as low-grade side reactions, while any symptoms of grade III or higher are classified as high-grade reactions (47). Overall, six studies have reported adverse reactions in patients with meningiomas that were treated with carbon ions. Five studies reported detailed data, among which one described only the symptoms and severity of adverse reactions (44), including alopecia, skin erythema, conjunctivitis, mucositis, dry mouth, headache, and nausea. As shown in **Table 3**, all side effects of CI-RT were grade I and grade II. Among them, focal alopecia, fatigue, skin stress and headache were the most common side effects of acute radiotherapy, with incidence rates of 19.5%, 15.3%, 10.5% and 10.1%, respectively. The results of the most common adverse reactions were almost the same proton and photon therapy, with incidence rates of 12%, 13%, 21% and 9%, respectively. With regards to the side effects of late radiotherapy, the most common adverse reactions were headache, sensory impairment, cognitive impairment, and hearing impairment, which had incidence rates of 19.4%, 23.7%, 9.1% and 9.1%, respectively.

**Table 3:** Acute and late treatment-related toxicity.

Intervention	Reason	Acute treatment-related toxicity ＜ 6 months		Rate (%)	Late treatment-related toxicity ＞ 6 months		Rate (%)
		CTCAE(I-II)	CTCAE (III OR higher)		CTCAE(I-II)	CTCAE (III OR higher)	
**Carbon+** **proton/photon**	Focal alopecia	117	0	19.5	2	0	0.9
	Fatigue	92	0	15.3	19	1	8.6
	Skin irritation	63	0	10.5	4	0	1.8
	Headache	61	0	10.1	45	0	19.4
	Nausea	42	1	7.2	8	0	3.4
	Localized pain	26	0	4.3	12	0	5.2
	Sensory deficits	72	0	12	55	0	23.7
	Lymphedema	9	0	1.5	6	0	2.6
	Xerostomia	6	0	0.9	6	1	3.2
	Mucositis	2	3	0.8	1	0	0.4
	Radio necrosis	1	0	0.2	0	3	1.3
	Cognitive dysfunction	16	0	2.7	21	0	9.1
	Hair loss	40	1	6.8	9	0	3.9
	Hearing impairment	16	1	2.8	21	0	9.1
	Dizziness	17	0	2.8	7	0	3
	Seizures	9	0	1.5	10	0	4
	Change in character	1	0	0.2	0	0	0
	Lacrimation of eyes	0	1	0.2	0	0	0
	Acute hemorrhage	0	0	0	1	0	0.4
	Slight visual impairment	4	0	0.7	0	0	0
	Total	594	7	100	227	5	100

**Proton**	Seizure	17	0	29.7	0	0	0
	Skin toxicity	26	0	45.1	0	0	0
	Optic neuropathy	4	0	6.8	1	0	16.7
	Dry eye	6	0	10.3	1	0	16.7
	Hypogonadism	3	0	5.2	2	0	33.3
	Asymptomatic hypothyroidism	2	0	3	2	0	33.3
	Total	58	0	100	6	0	100

**Proton+photon**	Vision loss	3	0	2	3	4	5.6
	Visual field deficit	1	0	1	6	0	4.8
	Diplopia	2	0	1	3	1	3.2
	Exophthalmos	1	0	1	2	0	2.5
	Conjunctivitis	3	0	2	0	0	0
	Eye, other	7		2	5	0	4
	Hearing loss	3	0	2	8	2	7.5
	Tinnitus	1	0	1	5	0	4
	Olfactory alteration	2	0	1	2	0	1.6
	Gustation alteration	4	0	2	1	0	0.8
	Dysphasia	0	0	0	1	0	0.8
	Neuromotor deficit	0	1	1	0	2	1.6
	Weakness	0	1	1	3	0	2.4
	Facial numbness	3	0	2	5	0	4
	Facial weakness	4	0	2	6	0	2.4
	Ataxia	1	0	1	5	0	4
	Seizure	2	0	1	0	0	0
	Fall	0	0	0	3	0	2.4
	Dysarthria	0	0	0	1	0	0.8
	Headache	11	0	9	8	0	6.4
	Nausea	17	0	12	0	0	0
	Dizziness	2	0	1	1	0	0.8
	Vertigo	5	0	2	2	0	2.4
	Syncope	1	0	1	0	0	0
	Depression	3	0	3	1	0	0.8
	Neuralog deficit	4	0	2	12	1	10.4
	Endocrine deficit	1	0	1	16	1	13.6
	Osteoporosis	0	0	0	1	0	0.8
	Cerebral edema	0	0	0	0	2	2.4
	Brain atrophy	0	0	0	1	0	0.8
	Skin changes	34	0	21	2	0	2.4
	Alopecia	18	0	12	2	0	2.4
	Fatigue	21	1	13	7	0	4.4
	Total	154	3	100	112	13	100


## Discussion

Our study aims to investigate the efficacy and safety, as well as the influencing factor of CI-RT among meningiomas. The results indicated that the OS, LC, and the common toxic and side effect of CI-RT for meningiomas is similar to PR-RT, better than PH-RT.

According to EANO (European Association of Neuro-Oncology) guidelines (48), for WHO grade I meningiomas that were totally resected, the 10-year recurrence varies from 20% to 39% (49–51). The 5-year progression of WHO grade II meningiomas may be as high as 30% after gross total resection and 40% after subtotal resection (52, 53). For WHO grade III meningiomas, the 5-year progression-free survival ranged from 12% to 57%, even after resection and radiotherapy (54). With the development of science and technology, radiotherapy for meningioma has been proven to be a promising treatment option, which is more effective than conventional surgical excision (55). Previous studies have reported local control rates ranging from 66.5% for grade II meningiomas at 2 years follow-up to 81% at 5 years for high-grade meningiomas using precision photon therapy (9). Same as our results shown in **Table 2**, the 5-year LC reached 68% to 87%, and the 5-year OS increased to 66% to 85% after particle RT. Among them, carbon-ion beams and protons directly cleave double-stranded DNA at low concentrations of oxygen and emit lower doses of radiation to the surrounding healthy tissue, which results in improved therapeutic ratios when compared to photon (56). A review by Adeberg et al. (28) supported the efficacy and safety of proton and carbon ion therapy. Consistent with our results, either CI-RT or PR-RT produced a better comparable rate of LC compared with traditional photon therapy. However, a recent systematic review presented comparable rates of LC between photon and proton RT with regards to benign brain tumors. Due to the small sample sizes, the conclusions may not robust enough (57). CI-RT for meningioma is a novel treatment. The inherent physical characteristics of CI-RT provide a special dose distribution, according to the specific range shown by Bragg Peak. This has the advantages of accuracy and omits key intracranial tissues, which make it particularly suitable for the treatment of these tumors (56). A systematic review by *Coggins et al.* indicated that ion therapy represents a burgeoning field in the treatment of atypical and anaplastic meningiomas. Proton and carbon ion radiotherapy maintain comparable rates of local control to conventional photon therapy and allow for more targeted treatment plans that may limit excess radiation damage (9). Although the Hug *et al.* study did not specify the rate of OS, the LC rate after protons and photons treatment (62 Gy E) was 88% (34), which was slightly better than CI-RT combined proton therapy. Regardless of whether the meningiomas are primary or recurrent, our meta-analysis also indicated that the LC of meningioma in 3- and 5-year after PR-RT has similar rates with CI-RT, and significantly better than PH-RT.

Treatment optimization for patients with high-grade meningiomas is the main goal for a radiation oncologist. It is known, that, for long-term local tumor control, high doses of radiotherapy are required (28). Previous studies have demonstrated beneficial results for particle therapy among patients with meningiomas (9, 58). However, most studies have evaluated PR-RT in low-grade meningioma patients (9, 57).Regarding differences in grade of meningioma, we observed superior local control over longer intended times of follow-up for grade II meningiomas. This finding remains unsurprising given the nature of histologic grading. Compared with CI-RT, we observed higher LC with PR-RT, which had a mean LC of 59.62% over 5 years. In contrast, CI-RT failed to deliver comparable rates of local control in either grade II (50% at 34 months) or III (63% at 2 years) meningiomas. However, our finding may not represent a deficiency between the two modalities and may be a result of heterogenous populations or patient selection factors. There was a protocol for the MARCIE trial with a carbon ion boost in combination with postoperative photon radiotherapy for Simpson grade 4 and 5 atypical meningiomas patients (46), and more trials are expected to be published in the future to produce more convincing results.

Five studies (28, 30, 31, 44, 45) reported minimal or no acute high-grade toxicities. However, three studies did report similar findings with regards to late high-grade toxicities (30, 31, 43). Furthermore, a study by El Shafie *et al.* (31), which has the highest sample size across all studies in our review, supports these results. This finding is highly significant as it corroborates the commonly held view that CI-RT has reduced side effects compared to conventional PH-RT (59). Most articles reported headaches and sensory impairment as the predominant adverse effect among patients, which is expected. The lack of late high-grade toxicities remains particularly promising as it affirms the hypothesis that CI-RT limits extraneous radiation to normal brain tissue (60, 61). These results are promising in confirming the belief that ion RT predisposes patients to marginal side effects.

Although this systematic review and meta-analysis has been proven to be an effective treatment against meningioma, our outcomes need to be treated with caution due to several significant limitations. Firstly, the number of studies included in this meta-analysis was not much many that some subgroup analyses could only be combined with two or three studies. As there is an obvious correlation between the study quality and results, this problem needs to be taken seriously. Secondly, all CI-RT studies were found to be from the same country, and heterogeneity among the studies was obvious. Hence, the bias of results could not be ruled out. Thirdly, many studies did not classify benign and non-benign meningiomas, which may confuse the conclusions. Although CI-RT is a novel clinical treatment, as it becomes more common and affordable, additional prospective studies with larger sample sizes will be necessary to quantify efficacy.

## Conclusions

CI-RT is a rapidly developing technique that has been proven to be an effective treatment against meningioma. The efficacy and safety of CI-RT for meningiomas is similar to PR-RT, better than PH-RT. However, there is a need for more prospective trials in order to quantify the efficacy of ion beam RT compared to conventional therapies and to provide meaningful comparisons of local control rates and survival rates among patients undergoing alternative interventions.

## Data Availability Statement

The original contributions presented in the study are included in the article/**Supplementary Material**, further inquiries can be directed to the corresponding authors.

## Author Contributions

Conception and design: K-HY, X-HW, and X-XL. Search and collection of data: J-YL, J-WL, M-XL. Data analysis and interpretation: L-PG, Z-TB, and FB. Manuscript writing: J-YL, J-WL, and Y-CJ. All authors contributed to the article and approved the submitted version.

## Funding

Supported by the National Social Science Fund of China (no. 19ZDA142); Key Laboratory of Evidence Based Medicine and Knowledge Translation Foundation of Gansu Province (no. GSEBMKT-2020KF01).

## Conflict of Interest

The authors declare that the research was conducted in the absence of any commercial or financial relationships that could be construed as a potential conflict of interest.
