# Supplementary material for: The Efficacy and Safety of Carbon Ion Radiotherapy for Meningiomas: A Systematic Review and Meta-Analysis
Source: Front Oncol. 2021 May 25;11:620534. doi: 10.3389/fonc.2021.620534 (PMC8185343; doi:10.3389/fonc.2021.620534)
Supplement: Supplementary file 1 [file Table_1.docx]

**Supplementary Material**


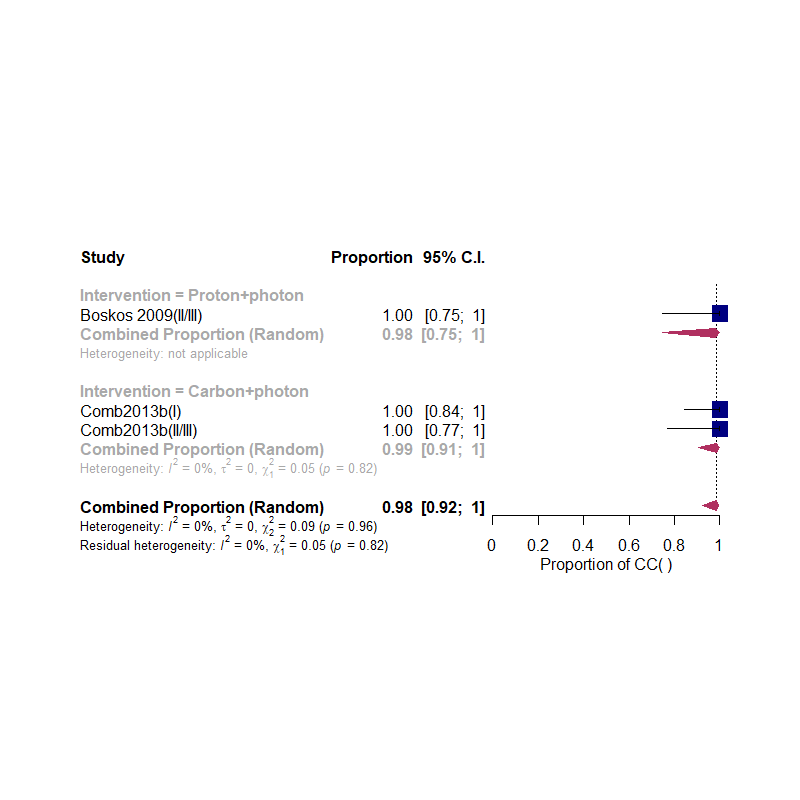


e-Fig.1 1-year overall survival rate after CI-RT, PR-RT, or PH-RT treatment


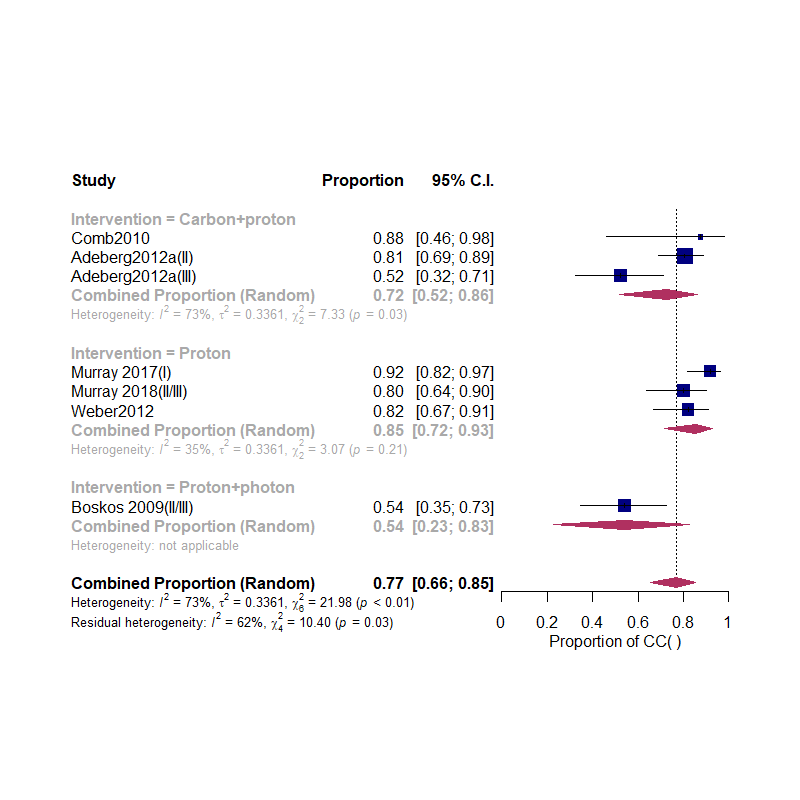


e-Fig.2 5-year overall survival rate after CI-RT, PR-RT, or PH-RT treatment


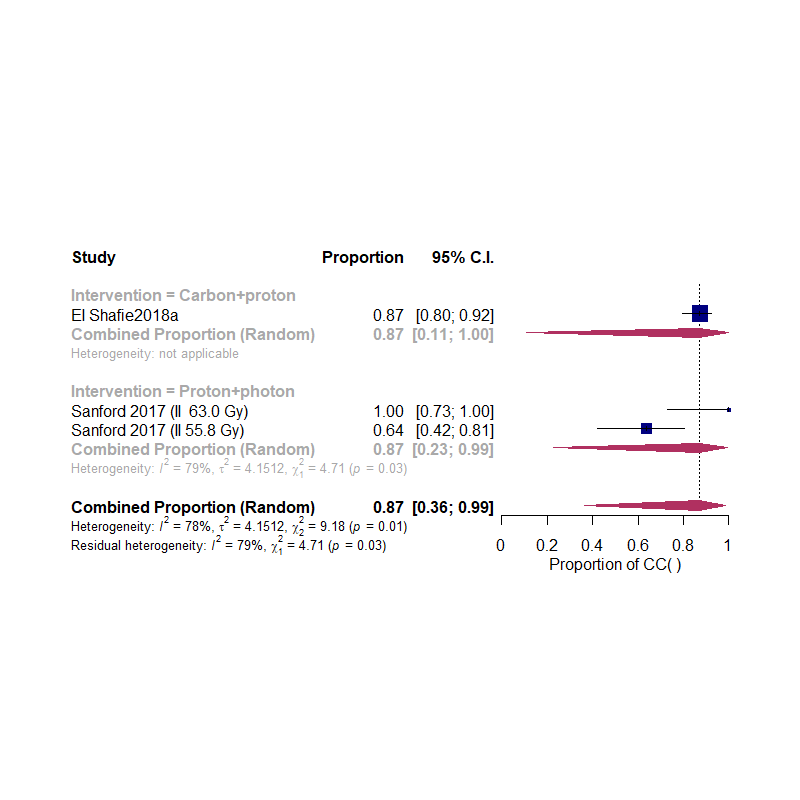


e-Fig.3 15-year overall survival rate after CI-RT, PR-RT, or PH-RT treatment


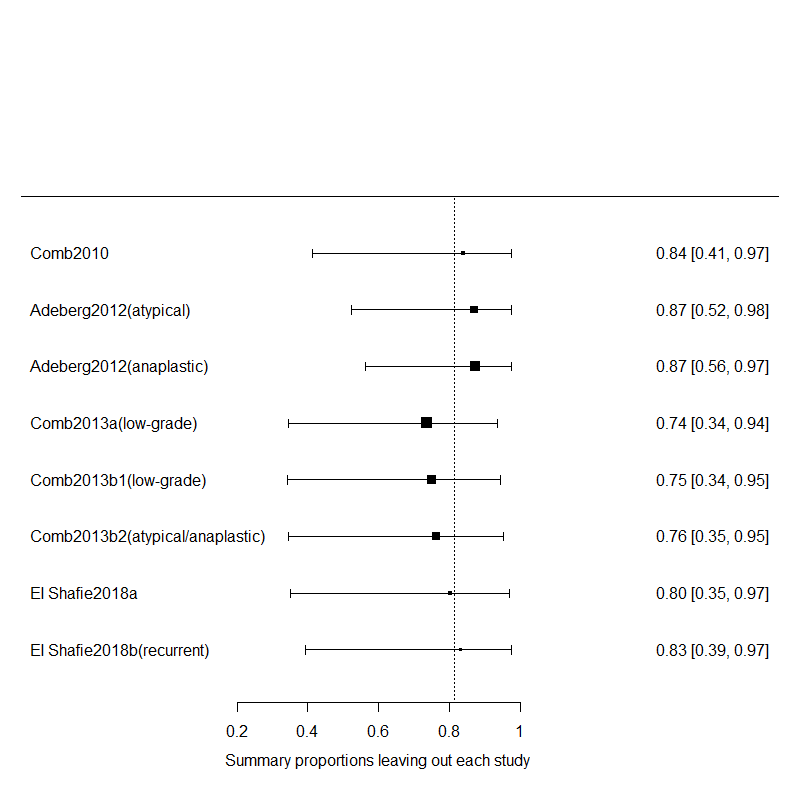


e-Fig.4. The result of sensitivity analysis


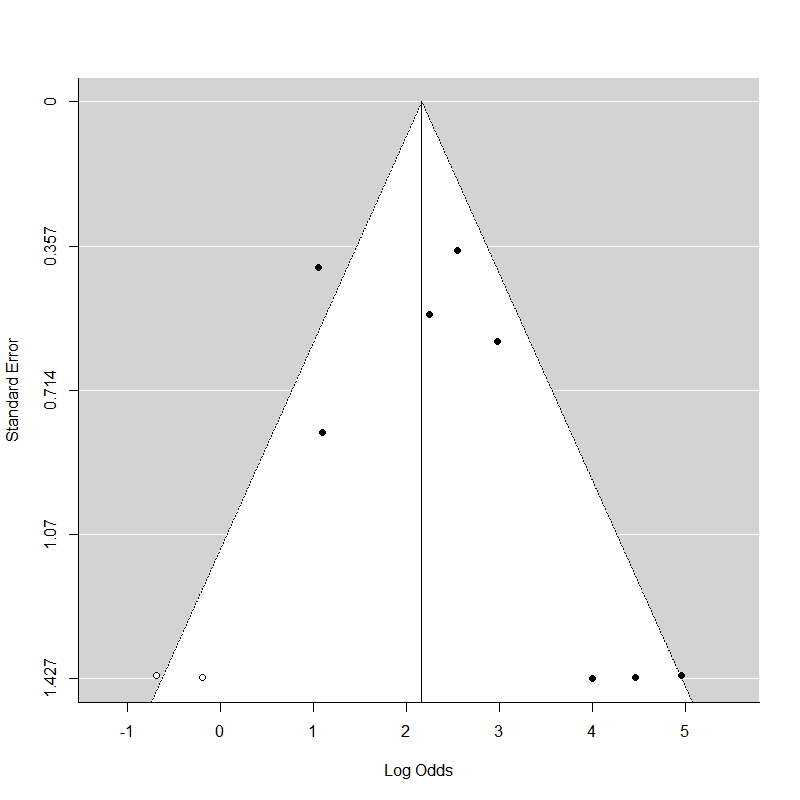


e-Fig.5 The trim and -fill funnel plot.


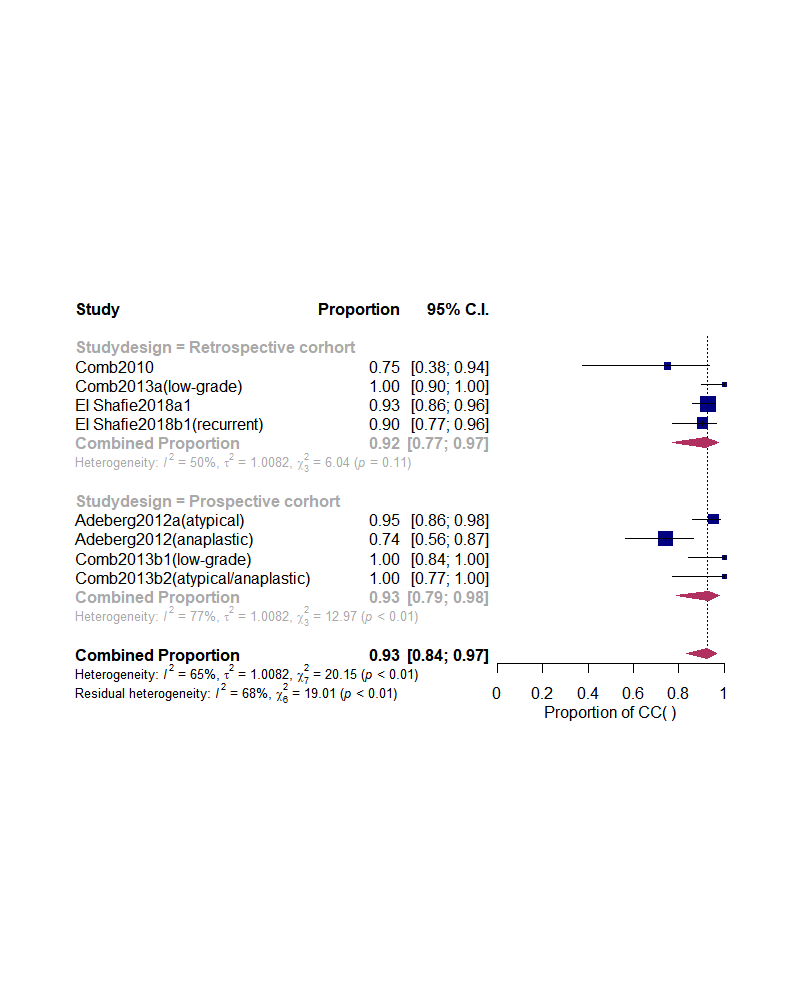


e-Fig.6 Association between prospective and retrospective cohort with the effectiveness of meningioma


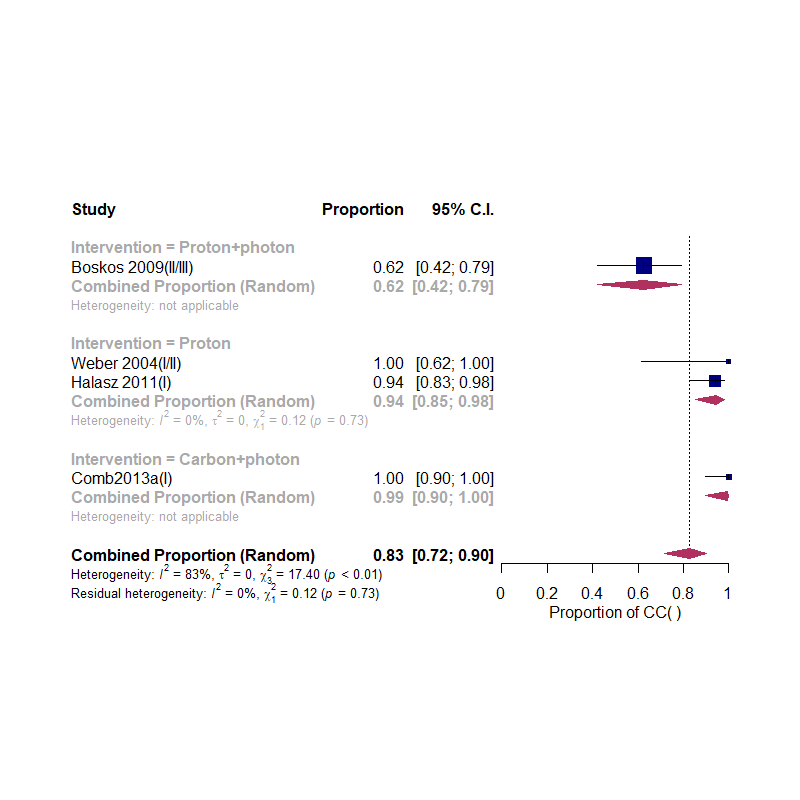


e-Fig.7 3-year local control rate after CI-RT, PR-RT, or PH-RT treatment


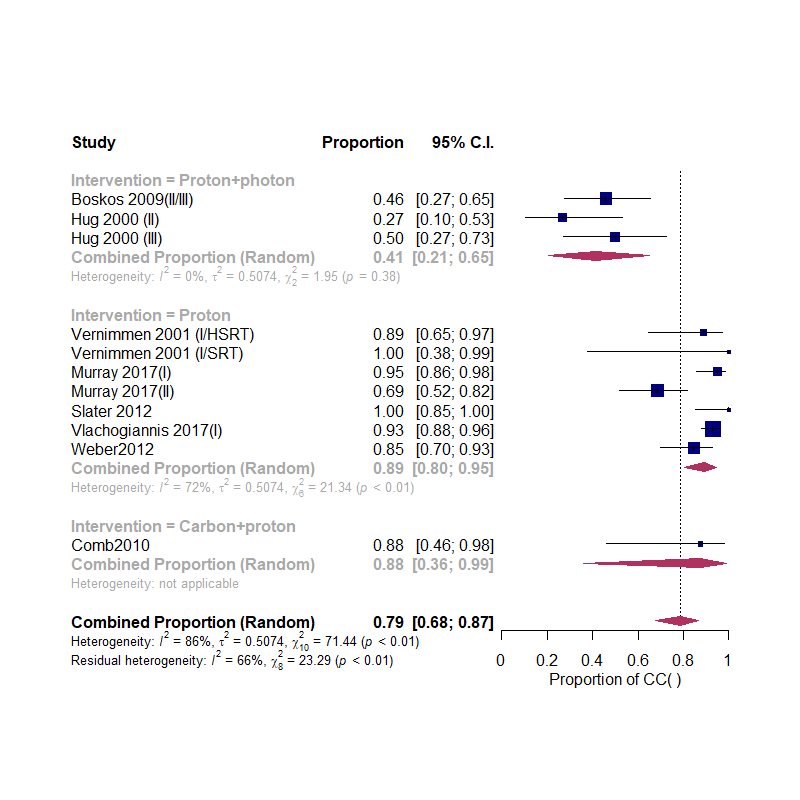


e-Fig.8 5-year local control rate after CI-RT, PR-RT, or PH-RT treatment

**R codes**

**Sensitivity analysis**

**> shuju <- read_excel("C:/Users/guolp/Desktop/shuju.xlsx",**

**+ sheet = "sen")**

**> View(shuju)**

**> ies.logit=escalc(xi=Number,ni=Total,data=shuju, measure = "PLO")**

**> pes.logit=rma(yi,vi,data = ies.logit)**

**> pes=predict(pes.logit,transf=transf.ilogit)**

**> ransf=transf.ilogit#inverse of logit transformation**

**> print(pes)**

**pred ci.lb ci.ub cr.lb cr.ub**

**0.8126 0.4348 0.9607 0.0318 0.9983**

**> print(pes.logit, digits=4)**

**Random-Effects Model (k = 8; tau^2 estimator: REML)**

**tau^2 (estimated amount of total heterogeneity): 5.4264 (SE = 3.3104)**

**tau (square root of estimated tau^2 value): 2.3295**

**I^2 (total heterogeneity / total variability): 95.49%**

**H^2 (total variability / sampling variability): 22.16**

**Test for Heterogeneity:**

**Q(df = 7) = 100.0032, p-val < .0001**

**Model Results:**

**estimate se zval pval ci.lb ci.ub**

**1.4667 0.8821 1.6628 0.0964 -0.2621 3.1955 .**

**---**

**Signif. codes: 0 ‘***’ 0.001 ‘**’ 0.01 ‘*’ 0.05 ‘.’ 0.1 ‘ ’ 1**

**>**

**> M=leave1out(pes.logit)**

**> yi=M$estimate; vi=M$se^2**

**> s1=forest(yi, vi, transf=transf.ilogit,**

**+ slab=paste(shuju$Study),**

**+ refline=pes$pred,**

**+ xlab="Summary proportions leaving out each study")**

**>**

**> fsn(yi, vi, data= ies.logit, type= "Rosenberg")**

**Fail-safe N Calculation Using the Rosenberg Approach**

**Average Effect Size: 0.5434**

**Observed Significance Level: 0.0011**

**Target Significance Level: 0.05**

**Fail-safe N: 15**

**> funnel(pes.logit)**

**> tf1=trimfill(pes.logit)**

**> funnel(tf1)**

**Short**

**> shuju <- read_excel("C:/Users/guolp/Desktop/shuju.xlsx",**

**+ sheet = "sen1")**

**> View(shuju)**

**> ies.logit=escalc(xi=Number,ni=Total,data=shuju, measure = "PLO")**

**> pes.logit=rma(yi,vi,data = ies.logit)**

**> pes=predict(pes.logit,transf=transf.ilogit)**

**> ransf=transf.ilogit#inverse of logit transformation**

**> print(pes)**

**pred ci.lb ci.ub cr.lb cr.ub**

**0.9229 0.8394 0.9648 0.6299 0.9883**

**> print(pes.logit, digits=4)**

**Random-Effects Model (k = 8; tau^2 estimator: REML)**

**tau^2 (estimated amount of total heterogeneity): 0.8122 (SE = 0.7280)**

**tau (square root of estimated tau^2 value): 0.9012**

**I^2 (total heterogeneity / total variability): 67.37%**

**H^2 (total variability / sampling variability): 3.06**

**Test for Heterogeneity:**

**Q(df = 7) = 20.1460, p-val = 0.0053**

**Model Results:**

**estimate se zval pval ci.lb ci.ub**

**2.4831 0.4230 5.8699 <.0001 1.6540 3.3122 *****

**---**

**Signif. codes: 0 ‘***’ 0.001 ‘**’ 0.01 ‘*’ 0.05 ‘.’ 0.1 ‘ ’ 1**

**>**

**> M=leave1out(pes.logit)**

**> yi=M$estimate; vi=M$se^2**

**> s1=forest(yi, vi, transf=transf.ilogit,**

**+ slab=paste(shuju$Study),**

**+ refline=pes$pred,**

**+ xlab="Summary proportions leaving out each study")**

**>**

**> fsn(yi, vi, data= ies.logit, type= "Rosenberg")**

**Fail-safe N Calculation Using the Rosenberg Approach**

**Average Effect Size: 2.1961**

**Observed Significance Level: <.0001**

**Target Significance Level: 0.05**

**Fail-safe N: 222**

**> funnel(pes.logit)**

**> tf1=trimfill(pes.logit)**

**> funnel(tf1)**

**Subgroup analysis**

**> shuju <- read_excel("C:/Users/guolp/Desktop/shuju.xlsx",**

**+ sheet = "sen1")**

**> View(shuju)**

**> ies.logit=escalc(xi=Number,ni=Total,data=shuju, measure = "PLO")**

**> pes.logit=rma(yi,vi,data = ies.logit)**

**> pes=predict(pes.logit,transf=transf.ilogit)**

**> ransf=transf.ilogit#inverse of logit transformation**

**> subganal.Studydesign=rma(yi, vi, data=ies.logit, mods=~ Studydesign, method="DL")**

**> pes.summary=metaprop(Number, Total, Study, data=shuju,**

**+ sm="PLO",**

**+ method.tau="DL",**

**+ method.ci="NAsm",**

**+ byvar= Studydesign,**

**+ tau.common=TRUE,**

**+ tau.preset=sqrt(subganal.Studydesign$tau2))**

**> pes.summary**

**proportion 95%-CI %W(fixed) %W(random)**

**Comb2010 0.7500 [0.3771; 0.9370] 6.6 12.4**

**Adeberg2012a(atypical) 0.9516 [0.8604; 0.9843] 12.5 15.3**

**Adeberg2012(anaplastic) 0.7419 [0.5626; 0.8654] 26.0 17.6**

**Comb2013a(low-grade) 1.0000 [0.8986; 0.9996] 2.2 6.9**

**Comb2013b1(low-grade) 1.0000 [0.8427; 0.9993] 2.2 6.8**

**Comb2013b2(atypical/anaplastic) 1.0000 [0.7704; 0.9989] 2.2 6.8**

**EI Shafie2018a1 0.9273 [0.8613; 0.9632] 32.5 18.1**

**EI Shafie2018b1(recurrent) 0.9048 [0.7722; 0.9638] 15.9 16.1**

**Studydesign**

**Comb2010 Retrospective corhort**

**Adeberg2012a(atypical) Prospective corhort**

**Adeberg2012(anaplastic) Prospective corhort**

**Comb2013a(low-grade) Retrospective corhort**

**Comb2013b1(low-grade) Prospective corhort**

**Comb2013b2(atypical/anaplastic) Prospective corhort**

**EI Shafie2018a1 Retrospective corhort**

**EI Shafie2018b1(recurrent) Retrospective corhort**

**Number of studies combined: k = 8**

**proportion 95%-CI**

**Fixed effect model 0.8999 [0.8564; 0.9313]**

**Random effects model 0.9255 [0.8358; 0.9681]**

**Quantifying heterogeneity:**

**tau^2 = 1.0082; tau = 1.0041; I^2 = 65.3% [26.0%; 83.7%]; H = 1.70 [1.16; 2.48]**

**Quantifying residual heterogeneity:**

**tau^2 = 1.0082; tau = 1.0041; I^2 = 68.4% [30.2%; 85.7%]; H = 1.78 [1.20; 2.65]**

**Test of heterogeneity:**

**Q d.f. p-value**

**20.15 7 0.0053**

**Results for subgroups (fixed effect model):**

**k proportion 95%-CI Q I^2**

**Studydesign = Retrospective corhort 4 0.9160 [0.8637; 0.9494] 6.04 50.3%**

**Studydesign = Prospective corhort 4 0.8742 [0.7877; 0.9286] 12.97 76.9%**

**Test for subgroup differences (fixed effect model):**

**Q d.f. p-value**

**Between groups 1.14 1 0.2863**

**Within groups 19.01 6 0.0041**

**Results for subgroups (random effects model):**

**k proportion 95%-CI tau^2 tau**

**Studydesign = Retrospective corhort 4 0.9192 [0.7707; 0.9747] 1.0082 1.0041**

**Studydesign = Prospective corhort 4 0.9321 [0.7878; 0.9807] 1.0082 1.0041**

**Test for subgroup differences (random effects model):**

**Q d.f. p-value**

**Between groups 0.04 1 0.8369**

**Details on meta-analytical method:**

**- Inverse variance method**

**- Preset square root of between-study variance: tau = 1.0041**

**- Logit transformation**

**- Normal approximation confidence interval for individual studies**

**- Continuity correction of 0.5 in studies with zero cell frequencies**

**> forest(pes.summary,**

**+ xlim=c(0,1),**

**+ rightcols=FALSE,**

**+ leftcols=c("studlab", "effect", "ci"),**

**+ leftlabs=c("Study", "Proportion", "95% C.I."),**

**+ text.random="Combined Proportion",**

**+ xlab="Proportion of CC( )", smlab="",**

**+ weight.study="fixed", col.square="navy",**

**+ col.diamond="maroon", col.diamond.lines="maroon",**

**+ fs.hetstat=10,**

**+ pooled.totals=FALSE,**

**+ comb.fixed=FALSE,**

**+ print.tau2=TRUE,**

**+ print.Q=TRUE,**

**+ print.pval.Q=TRUE,**

**+ print.I2=TRUE,**

**+ digits=2)**

**Forest-total**

**> shuju1 <- read_excel("C:/Users/guolp/Desktop/shuju1.xlsx",**

**+ sheet = "total")**

**> View(shuju1)**

**>**

**> ies.logit=escalc(xi=Number,ni=Total,data=shuju1, measure = "PLO")**

**> pes.logit=rma(yi,vi,data = ies.logit)**

**> pes=predict(pes.logit,transf=transf.ilogit)**

**> ransf=transf.ilogit#inverse of logit transformation**

**> subganal.OS=rma(yi, vi, data=ies.logit, mods=~ OS, method="DL")**

**> pes.summary=metaprop(Number, Total, Study, data=shuju1,**

**+ sm="PLO",**

**+ method.tau="DL",**

**+ method.ci="NAsm",**

**+ byvar= OS,**

**+ tau.common=TRUE,**

**+ tau.preset=sqrt(subganal.OS$tau2))**

**> pes.summary**

**proportion 95%-CI %W(fixed) %W(random) OS**

**Comb2013b(low-grade) 1.0000 [0.8427; 0.9993] 1.3 4.8 1years**

**Comb2013b(atypical/anaplastic) 1.0000 [0.7704; 0.9989] 1.3 4.8 1years**

**Comb2013a(low-grade) 1.0000 [0.8986; 0.9996] 1.3 4.9 3years**

**Comb2010 0.8750 [0.4627; 0.9827] 2.4 7.3 5years**

**Comb2010 0.7500 [0.3771; 0.9370] 4.0 10.0 10years**

**Adeberg2012a(atypical) 0.8065 [0.6893; 0.8867] 26.0 17.7 5years**

**Adeberg2012a(anaplastic) 0.5217 [0.3249; 0.7120] 15.4 16.1 5years**

**EI Shafie2018a 0.9455 [0.8839; 0.9753] 15.3 16.1 10years**

**EI Shafie2018a 0.8727 [0.7965; 0.9232] 32.9 18.3 15years**

**Number of studies combined: k = 9**

**proportion 95%-CI**

**Fixed effect model 0.8484 [0.8023; 0.8853]**

**Random effects model 0.8787 [0.7822; 0.9360]**

**Quantifying heterogeneity:**

**tau^2 = 0.6199; tau = 0.7873; I^2 = 77.8% [58.0%; 88.3%]; H = 2.12 [1.54; 2.92]**

**Quantifying residual heterogeneity:**

**tau^2 = 0.6199; tau = 0.7873; I^2 = 63.7% [4.4%; 86.2%]; H = 1.66 [1.02; 2.70]**

**Test of heterogeneity:**

**Q d.f. p-value**

**36.07 8 < 0.0001**

**Results for subgroups (fixed effect model):**

**k proportion 95%-CI Q I^2**

**OS = 1years 2 0.9858 [0.9058; 0.9980] 0.05 0.0%**

**OS = 3years 1 0.9931 [0.8986; 0.9996] 0.00 --**

**OS = 5years 3 0.7277 [0.6218; 0.8128] 7.33 72.7%**

**OS = 10years 2 0.9231 [0.8525; 0.9615] 3.65 72.6%**

**OS = 15years 1 0.8727 [0.7965; 0.9232] 0.00 --**

**Test for subgroup differences (fixed effect model):**

**Q d.f. p-value**

**Between groups 25.04 4 < 0.0001**

**Within groups 11.03 4 0.0262**

**Results for subgroups (random effects model):**

**k proportion 95%-CI tau^2 tau**

**OS = 1years 2 0.9858 [0.8788; 0.9985] 0.6199 0.7873**

**OS = 3years 1 0.9931 [0.8559; 0.9997] -- --**

**OS = 5years 3 0.7297 [0.4748; 0.8896] 0.6199 0.7873**

**OS = 10years 2 0.8986 [0.6916; 0.9723] 0.6199 0.7873**

**OS = 15years 1 0.8727 [0.5704; 0.9725] -- --**

**Test for subgroup differences (random effects model):**

**Q d.f. p-value**

**Between groups 10.44 4 0.0337**

**Details on meta-analytical method:**

**- Inverse variance method**

**- Preset square root of between-study variance: tau = 0.7873**

**- Logit transformation**

**- Normal approximation confidence interval for individual studies**

**- Continuity correction of 0.5 in studies with zero cell frequencies**

**> forest(pes.summary,**

**+ xlim=c(0,1),**

**+ rightcols=FALSE,**

**+ leftcols=c("studlab", "effect", "ci"),**

**+ leftlabs=c("Study", "Proportion", "95% C.I."),**

**text.random="Combined Proportion (Random)",**

**+ xlab="Proportion of CC( )", smlab="",**

**+ weight.study="fixed", col.square="navy",**

**+ col.diamond="maroon", col.diamond.lines="maroon",**

**+ fs.hetstat=10,**

**+ pooled.totals=FALSE,**

**+ comb.fixed=FALSE,**

**+ print.tau2=TRUE,**

**+ print.Q=TRUE,**

**+ print.pval.Q=TRUE,**

**+ print.I2=TRUE,**

**+ digits=2)**

**OS1**

**> data <- read_excel("C:/Users/guolp/Desktop/data.xlsx",**

**+ sheet = "OS1")**

**> View(data)**

**> ies.logit=escalc(xi=Number,ni=Total,data=data, measure = "PLO")**

**> pes.logit=rma(yi,vi,data = ies.logit)**

**> pes=predict(pes.logit,transf=transf.ilogit)**

**> ransf=transf.ilogit#inverse of logit transformation**

**> subganal.Intervention=rma(yi, vi, data=ies.logit, mods=~ Intervention, method="DL")**

**> pes.summary=metaprop(Number, Total, Study, data=data,**

**+ sm="PLO",**

**+ method.tau="DL",**

**+ method.ci="NAsm",**

**+ byvar= Intervention,**

**+ tau.common=TRUE,**

**+ tau.preset=sqrt(subganal.Intervention$tau2))**

**> pes.summary**

**proportion 95%-CI %W(fixed) %W(random) Intervention**

**Boskos 2009(II/III) 1.0000 [0.7487; 0.9988] 33.2 33.2 Proton+photon**

**Comb2013b(I) 1.0000 [0.8427; 0.9993] 33.5 33.5 Carbon+photon**

**Comb2013b(II/III) 1.0000 [0.7704; 0.9989] 33.3 33.3 Carbon+photon**

**Number of studies combined: k = 3**

**proportion 95%-CI**

**Fixed effect model 0.9841 [0.9248; 0.9968]**

**Random effects model 0.9841 [0.9248; 0.9968]**

**Quantifying heterogeneity:**

**tau^2 = 0; tau = 0; I^2 = 0.0% [0.0%; 0.0%]; H = 1.00 [1.00; 1.00]**

**Quantifying residual heterogeneity:**

**tau^2 = 0; tau = 0; I^2 = 0.0%; H = 1.00**

**Test of heterogeneity:**

**Q d.f. p-value**

**0.09 2 0.9556**

**Results for subgroups (fixed effect model):**

**k proportion 95%-CI Q I^2**

**Intervention = Proton+photon 1 0.9800 [0.7487; 0.9988] 0.00 --**

**Intervention = Carbon+photon 2 0.9858 [0.9058; 0.9980] 0.05 0.0%**

**Test for subgroup differences (fixed effect model):**

**Q d.f. p-value**

**Between groups 0.04 1 0.8433**

**Within groups 0.05 1 0.8200**

**Results for subgroups (random effects model):**

**k proportion 95%-CI tau^2 tau**

**Intervention = Proton+photon 1 0.9800 [0.7487; 0.9988] -- --**

**Intervention = Carbon+photon 2 0.9858 [0.9058; 0.9980] 0 0**

**Test for subgroup differences (random effects model):**

**Q d.f. p-value**

**Between groups 0.04 1 0.8433**

**Details on meta-analytical method:**

**- Inverse variance method**

**- Preset square root of between-study variance: tau = 0**

**- Logit transformation**

**- Normal approximation confidence interval for individual studies**

**- Continuity correction of 0.5 in studies with zero cell frequencies**

**> forest(pes.summary,**

**+ xlim=c(0,1),**

**+ rightcols=FALSE,**

**+ leftcols=c("studlab", "effect", "ci"),**

**+ leftlabs=c("Study", "Proportion", "95% C.I."),**

**+ text.random="Combined Proportion (Random)",**

**+ xlab="Proportion of CC( )", smlab="",**

**+ weight.study="fixed", col.square="navy",**

**+ col.diamond="maroon", col.diamond.lines="maroon",**

**+ fs.hetstat=10,**

**+ pooled.totals=FALSE,**

**+ comb.fixed=FALSE,**

**+ print.tau2=TRUE,**

**+ print.Q=TRUE,**

**+ print.pval.Q=TRUE,**

**+ print.I2=TRUE,**

**+ digits=2)**

**OS5**

**> data <- read_excel("C:/Users/guolp/Desktop/data.xlsx",**

**+ sheet = "OS5")**

**> View(data)**

**> ies.logit=escalc(xi=Number,ni=Total,data=data, measure = "PLO")**

**> pes.logit=rma(yi,vi,data = ies.logit)**

**> pes=predict(pes.logit,transf=transf.ilogit)**

**> ransf=transf.ilogit#inverse of logit transformation**

**> subganal.Intervention=rma(yi, vi, data=ies.logit, mods=~ Intervention, method="DL")**

**> pes.summary=metaprop(Number, Total, Study, data=data,**

**+ sm="PLO",**

**+ method.tau="DL",**

**+ method.ci="NAsm",**

**+ byvar= Intervention,**

**+ tau.common=TRUE,**

**+ tau.preset=sqrt(subganal.Intervention$tau2))**

**> pes.summary**

**proportion 95%-CI %W(fixed) %W(random) Intervention**

**Comb2010 0.8750 [0.4627; 0.9827] 2.3 5.4 Carbon+proton**

**Adeberg2012a(II) 0.8065 [0.6893; 0.8867] 25.3 18.1 Carbon+proton**

**Adeberg2012a(III) 0.5217 [0.3249; 0.7120] 15.0 15.5 Carbon+proton**

**Murray 2017(I) 0.9180 [0.8177; 0.9655] 12.0 14.3 Proton**

**Murray 2018(II/III) 0.8000 [0.6360; 0.9015] 14.7 15.4 Proton**

**Weber2012 0.8205 [0.6686; 0.9119] 15.0 15.6 Proton**

**Boskos 2009(II/III) 0.5417 [0.3462; 0.7251] 15.6 15.7 Proton+photon**

**Number of studies combined: k = 7**

**proportion 95%-CI**

**Fixed effect model 0.7627 [0.7007; 0.8153]**

**Random effects model 0.7682 [0.6561; 0.8520]**

**Quantifying heterogeneity:**

**tau^2 = 0.3361; tau = 0.5798; I^2 = 72.7% [41.2%; 87.3%]; H = 1.91 [1.30; 2.81]**

**Quantifying residual heterogeneity:**

**tau^2 = 0.3361; tau = 0.5798; I^2 = 61.5% [0.0%; 85.5%]; H = 1.61 [1.00; 2.63]**

**Test of heterogeneity:**

**Q d.f. p-value**

**21.98 6 0.0012**

**Results for subgroups (fixed effect model):**

**k proportion 95%-CI Q I^2**

**Intervention = Carbon+proton 3 0.7277 [0.6218; 0.8128] 7.33 72.7%**

**Intervention = Proton 3 0.8495 [0.7756; 0.9022] 3.07 35.0%**

**Intervention = Proton+photon 1 0.5417 [0.3462; 0.7251] 0.00 --**

**Test for subgroup differences (fixed effect model):**

**Q d.f. p-value**

**Between groups 11.58 2 0.0031**

**Within groups 10.40 4 0.0342**

**Results for subgroups (random effects model):**

**k proportion 95%-CI tau^2 tau**

**Intervention = Carbon+proton 3 0.7239 [0.5199; 0.8639] 0.3361 0.5798**

**Intervention = Proton 3 0.8529 [0.7186; 0.9294] 0.3361 0.5798**

**Intervention = Proton+photon 1 0.5417 [0.2272; 0.8261] -- --**

**Test for subgroup differences (random effects model):**

**Q d.f. p-value**

**Between groups 4.17 2 0.1245**

**Details on meta-analytical method:**

**- Inverse variance method**

**- Preset square root of between-study variance: tau = 0.5798**

**- Logit transformation**

**- Normal approximation confidence interval for individual studies**

**> forest(pes.summary,**

**+ xlim=c(0,1),**

**+ rightcols=FALSE,**

**+ leftcols=c("studlab", "effect", "ci"),**

**+ leftlabs=c("Study", "Proportion", "95% C.I."),**

**+ text.random="Combined Proportion (Random)",**

**+ xlab="Proportion of CC( )", smlab="",**

**+ weight.study="fixed", col.square="navy",**

**+ col.diamond="maroon", col.diamond.lines="maroon",**

**+ fs.hetstat=10,**

**+ pooled.totals=FALSE,**

**+ comb.fixed=FALSE,**

**+ print.tau2=TRUE,**

**+ print.Q=TRUE,**

**+ print.pval.Q=TRUE,**

**+ print.I2=TRUE,**

**+ digits=2)**

**OS10**

**> ies.logit=escalc(xi=Number,ni=Total,data=data, measure = "PLO")**

**> pes.logit=rma(yi,vi,data = ies.logit)**

**> pes=predict(pes.logit,transf=transf.ilogit)**

**> ransf=transf.ilogit#inverse of logit transformation**

**> print(pes)**

**pred ci.lb ci.ub cr.lb cr.ub**

**0.8924 0.6031 0.9784 0.3630 0.9918**

**> print(pes.logit, digits=4)**

**Random-Effects Model (k = 2; tau^2 estimator: REML)**

**tau^2 (estimated amount of total heterogeneity): 1.1168 (SE = 2.1755)**

**tau (square root of estimated tau^2 value): 1.0568**

**I^2 (total heterogeneity / total variability): 72.60%**

**H^2 (total variability / sampling variability): 3.65**

**Test for Heterogeneity:**

**Q(df = 1) = 3.6498, p-val = 0.0561**

**Model Results:**

**estimate se zval pval ci.lb ci.ub**

**2.1154 0.8658 2.4433 0.0146 0.4185 3.8123 ***

**---**

**Signif. codes: 0 ‘***’ 0.001 ‘**’ 0.01 ‘*’ 0.05 ‘.’ 0.1 ‘ ’ 1**

**OS15**

**> data <- read_excel("C:/Users/guolp/Desktop/data.xlsx",**

**+ sheet = "OS15")**

**> View(data)**

**>**

**> ies.logit=escalc(xi=Number,ni=Total,data=data, measure = "PLO")**

**> pes.logit=rma(yi,vi,data = ies.logit)**

**> pes=predict(pes.logit,transf=transf.ilogit)**

**> ransf=transf.ilogit#inverse of logit transformation**

**> subganal.Intervention=rma(yi, vi, data=ies.logit, mods=~ Intervention, method="DL")**

**> pes.summary=metaprop(Number, Total, Study, data=data,**

**+ sm="PLO",**

**+ method.tau="DL",**

**+ method.ci="NAsm",**

**+ byvar= Intervention,**

**+ tau.common=TRUE,**

**+ tau.preset=sqrt(subganal.Intervention$tau2))**

**> pes.summary**

**proportion 95%-CI %W(fixed) %W(random)**

**EI Shafie2018a 0.8727 [0.7965; 0.9232] 68.6 37.6**

**Sanford 2017 (II 63.0 Gy) 1.0000 [0.7319; 0.9987] 2.7 25.7**

**Sanford 2017 (II 55.8 Gy) 0.6364 [0.4233; 0.8066] 28.6 36.6**

**Intervention**

**EI Shafie2018a Carbon+proton**

**Sanford 2017 (II 63.0 Gy) Proton+photon**

**Sanford 2017 (II 55.8 Gy) Proton+photon**

**Number of studies combined: k = 3**

**proportion 95%-CI**

**Fixed effect model 0.8301 [0.7543; 0.8860]**

**Random effects model 0.8709 [0.3623; 0.9877]**

**Quantifying heterogeneity:**

**tau^2 = 4.1512; tau = 2.0375; I^2 = 78.2% [29.8%; 93.2%]; H = 2.14 [1.19; 3.85]**

**Quantifying residual heterogeneity:**

**tau^2 = 4.1512; tau = 2.0375; I^2 = 78.7% [7.7%; 95.1%]; H = 2.17 [1.04; 4.52]**

**Test of heterogeneity:**

**Q d.f. p-value**

**9.18 2 0.0101**

**Results for subgroups (fixed effect model):**

**k proportion 95%-CI Q I^2**

**Intervention = Carbon+proton 1 0.8727 [0.7965; 0.9232] 0.00 --**

**Intervention = Proton+photon 2 0.6994 [0.5036; 0.8421] 4.71 78.7%**

**Test for subgroup differences (fixed effect model):**

**Q d.f. p-value**

**Between groups 4.48 1 0.0344**

**Within groups 4.71 1 0.0301**

**Results for subgroups (random effects model):**

**k proportion 95%-CI tau^2 tau**

**Intervention = Carbon+proton 1 0.8727 [0.1084; 0.9974] -- --**

**Intervention = Proton+photon 2 0.8697 [0.2254; 0.9935] 4.1512 2.0375**

**Test for subgroup differences (random effects model):**

**Q d.f. p-value**

**Between groups 0.00 1 0.9918**

**Details on meta-analytical method:**

**- Inverse variance method**

**- Preset square root of between-study variance: tau = 2.0375**

**- Logit transformation**

**- Normal approximation confidence interval for individual studies**

**- Continuity correction of 0.5 in studies with zero cell frequencies**

**> forest(pes.summary,**

**+ xlim=c(0,1),**

**+ rightcols=FALSE,**

**+ leftcols=c("studlab", "effect", "ci"),**

**+ leftlabs=c("Study", "Proportion", "95% C.I."),**

**+ text.random="Combined Proportion (Random)",**

**+ xlab="Proportion of CC( )", smlab="",**

**+ weight.study="fixed", col.square="navy",**

**+ col.diamond="maroon", col.diamond.lines="maroon",**

**+ fs.hetstat=10,**

**+ pooled.totals=FALSE,**

**+ comb.fixed=FALSE,**

**+ print.tau2=TRUE,**

**+ print.Q=TRUE,**

**+ print.pval.Q=TRUE,**

**+ print.I2=TRUE,**

**+ digits=2)**

**LC1**

**data <- read_excel("C:/Users/guolp/Desktop/data1.xlsx",**

**sheet = "LC1")**

**View(data)**

**> ies.logit=escalc(xi=Number,ni=Total,data=data, measure = "PLO")**

**> pes.logit=rma(yi,vi,data = ies.logit)**

**> pes=predict(pes.logit,transf=transf.ilogit)**

**> ransf=transf.ilogit#inverse of logit transformation**

**> print(pes)**

**pred ci.lb ci.ub cr.lb cr.ub**

**0.6894 0.3395 0.9055 0.1705 0.9600**

**> print(pes.logit, digits=4)**

**Random-Effects Model (k = 2; tau^2 estimator: REML)**

**tau^2 (estimated amount of total heterogeneity): 0.9166 (SE = 1.5872)**

**tau (square root of estimated tau^2 value): 0.9574**

**I^2 (total heterogeneity / total variability): 81.67%**

**H^2 (total variability / sampling variability): 5.46**

**Test for Heterogeneity:**

**Q(df = 1) = 5.4554, p-val = 0.0195**

**Model Results:**

**estimate se zval pval ci.lb ci.ub**

**0.7974 0.7465 1.0683 0.2854 -0.6656 2.2604**

**---**

**Signif. codes: 0 ‘***’ 0.001 ‘**’ 0.01 ‘*’ 0.05 ‘.’ 0.1 ‘ ’ 1**

**> pes.summary=metaprop(Number, Total, Study, data=data, sm="PLO",**

**+ method.tau="DL", method.ci="NAsm")**

**> print(pes.summary)**

**proportion 95%-CI %W(fixed) %W(random)**

**Boskos 2009(II/III) 0.8333 [0.6309; 0.9360] 27.1 45.8**

**Comb2013a(II/III) 0.5278 [0.3675; 0.6826] 72.9 54.2**

**Number of studies combined: k = 2**

**proportion 95%-CI**

**Fixed effect model 0.6265 [0.4896; 0.7457]**

**Random effects model 0.6894 [0.3395; 0.9055]**

**Quantifying heterogeneity:**

**tau^2 = 0.9166; tau = 0.9574; I^2 = 81.7% [22.3%; 95.7%]; H = 2.34 [1.13; 4.81]**

**Test of heterogeneity:**

**Q d.f. p-value**

**5.46 1 0.0195**

**> forest(pes.summary,**

**+ xlim=c(0,1),**

**+ rightcols=FALSE,**

**+ leftcols=c("studlab", "effect", "ci"),**

**+ leftlabs=c("Study", "Proportion", "95% C.I."),**

**+ text.random="Combined Proportion (Random)",**

**+ xlab="Proportion of CC( )", smlab="",**

**+ weight.study="fixed", col.square="navy",**

**+ col.diamond="maroon", col.diamond.lines="maroon",**

**+ fs.hetstat=10,**

**+ pooled.totals=FALSE,**

**+ comb.fixed=FALSE,**

**+ print.tau2=TRUE,**

**+ print.Q=TRUE,**

**+ print.pval.Q=TRUE,**

**+ print.I2=TRUE,**

**+ digits=2)**

**OS3**

**> data <- read_excel("C:/Users/guolp/Desktop/data.xlsx",**

**+ sheet = "OS3")**

**> View(data)**

**> ies.logit=escalc(xi=Number,ni=Total,data=data, measure = "PLO")**

**> pes.logit=rma(yi,vi,data = ies.logit)**

**> pes=predict(pes.logit,transf=transf.ilogit)**

**> ransf=transf.ilogit#inverse of logit transformation**

**> print(pes)**

**pred ci.lb ci.ub cr.lb cr.ub**

**0.9079 0.6118 0.9840 0.2790 0.9960**

**> print(pes.logit, digits=4)**

**Random-Effects Model (k = 3; tau^2 estimator: REML)**

**tau^2 (estimated amount of total heterogeneity): 1.8536 (SE = 2.6537)**

**tau (square root of estimated tau^2 value): 1.3615**

**I^2 (total heterogeneity / total variability): 74.13%**

**H^2 (total variability / sampling variability): 3.87**

**Test for Heterogeneity:**

**Q(df = 2) = 5.8080, p-val = 0.0548**

**Model Results:**

**estimate se zval pval ci.lb ci.ub**

**2.2885 0.9356 2.4460 0.0144 0.4547 4.1222 ***

**---**

**Signif. codes: 0 ‘***’ 0.001 ‘**’ 0.01 ‘*’ 0.05 ‘.’ 0.1 ‘ ’ 1**

**> pes.summary=metaprop(Number, Total, Study, data=data, sm="PLO",**

**+ method.tau="DL", method.ci="NAsm")**

**> print(pes.summary)**

**proportion 95%-CI %W(fixed) %W(random)**

**Comb2013a(I)-Carbon+photon 1.0000 [0.8986; 0.9996] 8.1 20.1**

**Weber 2004(I/II)-Proton 0.8462 [0.5494; 0.9613] 27.5 35.8**

**Boskos 2009(II/III)-Proton+photon 0.7917 [0.5866; 0.9105] 64.4 44.0**

**Number of studies combined: k = 3**

**proportion 95%-CI**

**Fixed effect model 0.8494 [0.7190; 0.9256]**

**Random effects model 0.9001 [0.6488; 0.9777]**

**Quantifying heterogeneity:**

**tau^2 = 1.2315 [0.0000; >100.0000]; tau = 1.1097 [0.0000; >10.0000];**

**I^2 = 65.6% [0.0%; 90.1%]; H = 1.70 [1.00; 3.18]**

**Test of heterogeneity:**

**Q d.f. p-value**

**5.81 2 0.0548**

**Details on meta-analytical method:**

**- Inverse variance method**

**- DerSimonian-Laird estimator for tau^2**

**- Jackson method for confidence interval of tau^2 and tau**

**- Logit transformation**

**- Normal approximation confidence interval for individual studies**

**- Continuity correction of 0.5 in studies with zero cell frequencies**

**>**

**> forest(pes.summary,**

**+ xlim=c(0,1),**

**+ rightcols=FALSE,**

**+ leftcols=c("studlab", "effect", "ci"),**

**+ leftlabs=c("Study", "Proportion", "95% C.I."),**

**+ text.random="Combined Proportion (Random)",**

**+ xlab="Proportion of CC( )", smlab="",**

**+ weight.study="fixed", col.square="navy",**

**+ col.diamond="maroon", col.diamond.lines="maroon",**

**+ fs.hetstat=10,**

**+ pooled.totals=FALSE,**

**+ comb.fixed=FALSE,**

**+ print.tau2=TRUE,**

**+ print.Q=TRUE,**

**+ print.pval.Q=TRUE,**

**+ print.I2=TRUE,**

**+ digits=2)**

**LC2**

**> data <- read_excel("C:/Users/guolp/Desktop/data1.xlsx",**

**+ sheet = "LC2")**

**> View(data)**

**> ies.logit=escalc(xi=Number,ni=Total,data=data, measure = "PLO")**

**> pes.logit=rma(yi,vi,data = ies.logit)**

**> pes=predict(pes.logit,transf=transf.ilogit)**

**> ransf=transf.ilogit#inverse of logit transformation**

**> print(pes)**

**pred ci.lb ci.ub cr.lb cr.ub**

**0.6035 0.1376 0.9356 0.0329 0.9855**

**> print(pes.logit, digits=4)**

**Random-Effects Model (k = 2; tau^2 estimator: REML)**

**tau^2 (estimated amount of total heterogeneity): 2.4384 (SE = 3.7490)**

**tau (square root of estimated tau^2 value): 1.5616**

**I^2 (total heterogeneity / total variability): 91.98%**

**H^2 (total variability / sampling variability): 12.48**

**Test for Heterogeneity:**

**Q(df = 1) = 12.4751, p-val = 0.0004**

**Model Results:**

**estimate se zval pval ci.lb ci.ub**

**0.4201 1.1507 0.3651 0.7150 -1.8351 2.6754**

**---**

**Signif. codes: 0 ‘***’ 0.001 ‘**’ 0.01 ‘*’ 0.05 ‘.’ 0.1 ‘ ’ 1**

**> pes.summary=metaprop(Number, Total, Study, data=data, sm="PLO",**

**+ method.tau="DL", method.ci="NAsm")**

**> print(pes.summary)**

**proportion 95%-CI %W(fixed) %W(random)**

**Boskos 2009(II/III) 0.8333 [0.6309; 0.9360] 29.4 48.3**

**Comb2013a(II/III) 0.3333 [0.2000; 0.5000] 70.6 51.7**

**Number of studies combined: k = 2**

**proportion 95%-CI**

**Fixed effect model 0.4960 [0.3548; 0.6379]**

**Random effects model 0.6035 [0.1376; 0.9356]**

**Quantifying heterogeneity:**

**tau^2 = 2.4384; tau = 1.5616; I^2 = 92.0% [72.4%; 97.7%]; H = 3.53 [1.90; 6.56]**

**Test of heterogeneity:**

**Q d.f. p-value**

**12.48 1 0.0004**

**Details on meta-analytical method:**

**- Inverse variance method**

**- DerSimonian-Laird estimator for tau^2**

**- Logit transformation**

**- Normal approximation confidence interval for individual studies**

**>**

**> forest(pes.summary,**

**+ xlim=c(0,1),**

**+ rightcols=FALSE,**

**+ leftcols=c("studlab", "effect", "ci"),**

**+ leftlabs=c("Study", "Proportion", "95% C.I."),**

**+ text.random="Combined Proportion (Random)",**

**+ xlab="Proportion of CC( )", smlab="",**

**+ weight.study="fixed", col.square="navy",**

**+ col.diamond="maroon", col.diamond.lines="maroon",**

**+ fs.hetstat=10,**

**+ pooled.totals=FALSE,**

**+ comb.fixed=FALSE,**

**+ print.tau2=TRUE,**

**+ print.Q=TRUE,**

**+ print.pval.Q=TRUE,**

**+ print.I2=TRUE,**

**+ digits=2)**

**LC3**

**data <- read_excel("C:/Users/guolp/Desktop/data1.xlsx", sheet = "LC3")**

**> View(data)**

**> ies.logit=escalc(xi=Number,ni=Total,data=data, measure = "PLO")**

**> pes.logit=rma(yi,vi,data = ies.logit)**

**> pes=predict(pes.logit,transf=transf.ilogit)**

**> ransf=transf.ilogit#inverse of logit transformation**

**> subganal.Intervention=rma(yi, vi, data=ies.logit, mods=~ Intervention, method="DL")**

**> pes.summary=metaprop(Number, Total, Study, data=data,**

**+ sm="PLO",**

**+ method.tau="DL",**

**+ method.ci="NAsm",**

**+ byvar= Intervention,**

**+ tau.common=TRUE,**

**+ tau.preset=sqrt(subganal.Intervention$tau2))**

**> pes.summary**

**proportion 95%-CI %W(fixed) %W(random) Intervention**

**Boskos 2009(II/III) 0.6250 [0.4218; 0.7920] 59.7 59.7 Proton+photon**

**Weber 2004(I/II) 1.0000 [0.6161; 0.9978] 5.1 5.1 Proton**

**Halasz 2011(I) 0.9400 [0.8298; 0.9805] 29.9 29.9 Proton**

**Comb2013a(I) 1.0000 [0.8986; 0.9996] 5.3 5.3 Carbon+photon**

**Number of studies combined: k = 4**

**proportion 95%-CI**

**Fixed effect model 0.8261 [0.7150; 0.9000]**

**Random effects model 0.8261 [0.7150; 0.9000]**

**Quantifying heterogeneity:**

**tau^2 = 0; tau = 0; I^2 = 82.8% [55.8%; 93.3%]; H = 2.41 [1.50; 3.85]**

**Quantifying residual heterogeneity:**

**tau^2 = 0; tau = 0; I^2 = 0.0%; H = 1.00**

**Test of heterogeneity:**

**Q d.f. p-value**

**17.40 3 0.0006**

**Results for subgroups (fixed effect model):**

**k proportion 95%-CI Q I^2**

**Intervention = Proton+photon 1 0.6250 [0.4218; 0.7920] 0.00 --**

**Intervention = Proton 2 0.9443 [0.8523; 0.9803] 0.12 0.0%**

**Intervention = Carbon+photon 1 0.9931 [0.8986; 0.9996] 0.00 --**

**Test for subgroup differences (fixed effect model):**

**Q d.f. p-value**

**Between groups 17.28 2 0.0002**

**Within groups 0.12 1 0.7269**

**Results for subgroups (random effects model):**

**k proportion 95%-CI tau^2 tau**

**Intervention = Proton+photon 1 0.6250 [0.4218; 0.7920] -- --**

**Intervention = Proton 2 0.9443 [0.8523; 0.9803] 0 0**

**Intervention = Carbon+photon 1 0.9931 [0.8986; 0.9996] -- --**

**Test for subgroup differences (random effects model):**

**Q d.f. p-value**

**Between groups 17.28 2 0.0002**

**Details on meta-analytical method:**

**- Inverse variance method**

**- Preset square root of between-study variance: tau = 0**

**- Logit transformation**

**- Normal approximation confidence interval for individual studies**

**- Continuity correction of 0.5 in studies with zero cell frequencies**

**> forest(pes.summary,**

**+ xlim=c(0,1),**

**+ rightcols=FALSE,**

**+ leftcols=c("studlab", "effect", "ci"),**

**+ leftlabs=c("Study", "Proportion", "95% C.I."),**

**+ text.random="Combined Proportion (Random)",**

**+ xlab="Proportion of CC( )", smlab="",**

**+ weight.study="fixed", col.square="navy",**

**+ col.diamond="maroon", col.diamond.lines="maroon",**

**+ fs.hetstat=10,**

**+ pooled.totals=FALSE,**

**+ comb.fixed=FALSE,**

**+ print.tau2=TRUE,**

**+ print.Q=TRUE,**

**+ print.pval.Q=TRUE,**

**+ print.I2=TRUE,**

**+ digits=2)**

**LC5**

**> data <- read_excel("C:/Users/guolp/Desktop/data1.xlsx", sheet = "LC5")**

**> View(data)**

**> ies.logit=escalc(xi=Number,ni=Total,data=data, measure = "PLO")**

**> pes.logit=rma(yi,vi,data = ies.logit)**

**> pes=predict(pes.logit,transf=transf.ilogit)**

**> ransf=transf.ilogit#inverse of logit transformation**

**> subganal.Intervention=rma(yi, vi, data=ies.logit, mods=~ Intervention, method="DL")**

**> pes.summary=metaprop(Number, Total, Study, data=data,**

**+ sm="PLO",**

**+ method.tau="DL",**

**+ method.ci="NAsm",**

**+ byvar= Intervention,**

**+ tau.common=TRUE,**

**+ tau.preset=sqrt(subganal.Intervention$tau2))**

**> pes.summary**

**proportion 95%-CI %W(fixed) %W(random) Intervention**

**Boskos 2009(II/III) 0.4583 [0.2749; 0.6538] 13.8 12.2 Proton+photon**

**Hug 2000 (II) 0.2667 [0.1038; 0.5331] 6.8 9.7 Proton+photon**

**Hug 2000 (III) 0.5000 [0.2729; 0.7271] 9.3 10.9 Proton+photon**

**Vernimmen 2001 (I/HSRT) 0.8889 [0.6478; 0.9721] 4.1 7.7 Proton**

**Vernimmen 2001 (I/SRT) 1.0000 [0.3782; 0.9950] 1.1 3.1 Proton**

**Murray 2017(I) 0.9508 [0.8583; 0.9841] 6.6 9.6 Proton**

**Murray 2017(II) 0.6857 [0.5166; 0.8166] 17.5 12.9 Proton**

**Slater 2012 1.0000 [0.8541; 0.9994] 1.1 3.3 Proton**

**Vlachogiannis 2017(I) 0.9294 [0.8798; 0.9595] 25.9 13.8 Proton**

**Weber2012 0.8462 [0.6974; 0.9292] 11.8 11.7 Proton**

**Comb2010 0.8750 [0.4627; 0.9827] 2.0 5.0 Carbon+proton**

**Number of studies combined: k = 11**

**proportion 95%-CI**

**Fixed effect model 0.7876 [0.7335; 0.8333]**

**Random effects model 0.7864 [0.6770; 0.8661]**

**Quantifying heterogeneity:**

**tau^2 = 0.5074; tau = 0.7123; I^2 = 86.0% [76.8%; 91.6%]; H = 2.67 [2.07; 3.44]**

**Quantifying residual heterogeneity:**

**tau^2 = 0.5074; tau = 0.7123; I^2 = 65.6% [30.2%; 83.1%]; H = 1.71 [1.20; 2.43]**

**Test of heterogeneity:**

**Q d.f. p-value**

**71.44 10 < 0.0001**

**Results for subgroups (fixed effect model):**

**k proportion 95%-CI Q I^2**

**Intervention = Proton+photon 3 0.4238 [0.2987; 0.5593] 1.95 0.0%**

**Intervention = Proton 7 0.8811 [0.8376; 0.9141] 21.34 71.9%**

**Intervention = Carbon+proton 1 0.8750 [0.4627; 0.9827] 0.00 --**

**Test for subgroup differences (fixed effect model):**

**Q d.f. p-value**

**Between groups 48.16 2 < 0.0001**

**Within groups 23.29 8 0.0030**

**Results for subgroups (random effects model):**

**k proportion 95%-CI tau^2 tau**

**Intervention = Proton+photon 3 0.4105 [0.2068; 0.6504] 0.5074 0.7123**

**Intervention = Proton 7 0.8940 [0.8050; 0.9452] 0.5074 0.7123**

**Intervention = Carbon+proton 1 0.8750 [0.3608; 0.9886] -- --**

**Test for subgroup differences (random effects model):**

**Q d.f. p-value**

**Between groups 16.46 2 0.0003**

**Details on meta-analytical method:**

**- Inverse variance method**

**- Preset square root of between-study variance: tau = 0.7123**

**- Logit transformation**

**- Normal approximation confidence interval for individual studies**

**- Continuity correction of 0.5 in studies with zero cell frequencies**

**> forest(pes.summary,**

**+ xlim=c(0,1),**

**+ rightcols=FALSE,**

**+ leftcols=c("studlab", "effect", "ci"),**

**+ leftlabs=c("Study", "Proportion", "95% C.I."),**

**+ text.random="Combined Proportion (Random)",**

**+ xlab="Proportion of CC( )", smlab="",**

**+ weight.study="fixed", col.square="navy",**

**+ col.diamond="maroon", col.diamond.lines="maroon",**

**+ fs.hetstat=10,**

**+ pooled.totals=FALSE,**

**+ comb.fixed=FALSE,**

**+ print.tau2=TRUE,**

**+ print.Q=TRUE,**

**+ print.pval.Q=TRUE,**

**+ print.I2=TRUE,**

**+ digits=2)**

**LC8**

**data <- read_excel("C:/Users/guolp/Desktop/data1.xlsx",**

**+ sheet = "LC8")**

**> View(data)**

**> ies.logit=escalc(xi=Number,ni=Total,data=data, measure = "PLO")**

**> pes.logit=rma(yi,vi,data = ies.logit)**

**> pes=predict(pes.logit,transf=transf.ilogit)**

**> ransf=transf.ilogit#inverse of logit transformation**

**> print(pes)**

**pred ci.lb ci.ub cr.lb cr.ub**

**0.2909 0.1412 0.5059 0.0862 0.6407**

**> print(pes.logit, digits=4)**

**Random-Effects Model (k = 3; tau^2 estimator: REML)**

**tau^2 (estimated amount of total heterogeneity): 0.3443 (SE = 0.6611)**

**tau (square root of estimated tau^2 value): 0.5868**

**I^2 (total heterogeneity / total variability): 52.51%**

**H^2 (total variability / sampling variability): 2.11**

**Test for Heterogeneity:**

**Q(df = 2) = 4.2435, p-val = 0.1198**

**Model Results:**

**estimate se zval pval ci.lb ci.ub**

**-0.8909 0.4667 -1.9092 0.0562 -1.8056 0.0237 .**

**---**

**Signif. codes: 0 ‘***’ 0.001 ‘**’ 0.01 ‘*’ 0.05 ‘.’ 0.1 ‘ ’ 1**

**> pes.summary=metaprop(Number, Total, Study, data=data, sm="PLO",**

**+ method.tau="DL", method.ci="NAsm")**

**> print(pes.summary)**

**proportion 95%-CI %W(fixed) %W(random)**

**Boskos 2009(II/III) 0.4583 [0.2749; 0.6538] 55.2 42.4**

**Hug 2000 (II) 0.2000 [0.0659; 0.4698] 22.2 28.7**

**Hug 2000 (III) 0.1875 [0.0617; 0.4475] 22.6 28.9**

**Number of studies combined: k = 3**

**proportion 95%-CI**

**Fixed effect model 0.3249 [0.2095; 0.4663]**

**Random effects model 0.2907 [0.1406; 0.5066]**

**Quantifying heterogeneity:**

**tau^2 = 0.3493 [0.0000; 23.7056]; tau = 0.5910 [0.0000; 4.8688];**

**I^2 = 52.9% [0.0%; 86.5%]; H = 1.46 [1.00; 2.72]**

**Test of heterogeneity:**

**Q d.f. p-value**

**4.24 2 0.1198**

**Details on meta-analytical method:**

**- Inverse variance method**

**- DerSimonian-Laird estimator for tau^2**

**- Jackson method for confidence interval of tau^2 and tau**

**- Logit transformation**

**- Normal approximation confidence interval for individual studies**

**>**

**> forest(pes.summary,**

**+ xlim=c(0,1),**

**+ rightcols=FALSE,**

**+ leftcols=c("studlab", "effect", "ci"),**

**+ leftlabs=c("Study", "Proportion", "95% C.I."),**

**+ text.random="Combined Proportion (Random)",**

**+ xlab="Proportion of CC( )", smlab="",**

**+ weight.study="fixed", col.square="navy",**

**+ col.diamond="maroon", col.diamond.lines="maroon",**

**+ fs.hetstat=10,**

**+ pooled.totals=FALSE,**

**+ comb.fixed=FALSE,**

**+ print.tau2=TRUE,**

**+ print.Q=TRUE,**

**+ print.pval.Q=TRUE,**

**+ print.I2=TRUE,**

**+ digits=2)**

**LC10**

**> data <- read_excel("C:/Users/guolp/Desktop/data1.xlsx",**

**+ sheet = "LC10")**

**> View(data)**

**> ies.logit=escalc(xi=Number,ni=Total,data=data, measure = "PLO")**

**> pes.logit=rma(yi,vi,data = ies.logit)**

**> pes=predict(pes.logit,transf=transf.ilogit)**

**> ransf=transf.ilogit#inverse of logit transformation**

**> print(pes)**

**pred ci.lb ci.ub cr.lb cr.ub**

**0.9252 0.6483 0.9881 0.3704 0.9962**

**> print(pes.logit, digits=4)**

**Random-Effects Model (k = 2; tau^2 estimator: REML)**

**tau^2 (estimated amount of total heterogeneity): 1.4716 (SE = 2.8379)**

**tau (square root of estimated tau^2 value): 1.2131**

**I^2 (total heterogeneity / total variability): 73.34%**

**H^2 (total variability / sampling variability): 3.75**

**Test for Heterogeneity:**

**Q(df = 1) = 3.7503, p-val = 0.0528**

**Model Results:**

**estimate se zval pval ci.lb ci.ub**

**2.5158 0.9716 2.5894 0.0096 0.6116 4.4201 ****

**---**

**Signif. codes: 0 ‘***’ 0.001 ‘**’ 0.01 ‘*’ 0.05 ‘.’ 0.1 ‘ ’ 1**

**> pes.summary=metaprop(Number, Total, Study, data=data, sm="PLO",**

**+ method.tau="DL", method.ci="NAsm")**

**> print(pes.summary)**

**proportion 95%-CI %W(fixed) %W(random)**

**Sanford 2017-Proton+photon 0.9773 [0.8555; 0.9968] 4.4 37.8**

**Vlachogiannis 2017(I)-Proton 0.8529 [0.7914; 0.8986] 95.6 62.2**

**Number of studies combined: k = 2**

**proportion 95%-CI**

**Fixed effect model 0.8636 [0.8070; 0.9056]**

**Random effects model 0.9252 [0.6483; 0.9881]**

**Quantifying heterogeneity:**

**tau^2 = 1.4716; tau = 1.2131; I^2 = 73.3% [0.0%; 94.0%]; H = 1.94 [1.00; 4.08]**

**Test of heterogeneity:**

**Q d.f. p-value**

**3.75 1 0.0528**

**Details on meta-analytical method:**

**- Inverse variance method**

**- DerSimonian-Laird estimator for tau^2**

**- Logit transformation**

**- Normal approximation confidence interval for individual studies**

**>**

**> forest(pes.summary,**

**+ xlim=c(0,1),**

**+ rightcols=FALSE,**

**+ leftcols=c("studlab", "effect", "ci"),**

**+ leftlabs=c("Study", "Proportion", "95% C.I."),**

**+ text.random="Combined Proportion (Random)",**

**+ xlab="Proportion of CC( )", smlab="",**

**+ weight.study="fixed", col.square="navy",**

**+ col.diamond="maroon", col.diamond.lines="maroon",**

**+ fs.hetstat=10,**

**+ pooled.totals=FALSE,**

**+ comb.fixed=FALSE,**

**+ print.tau2=TRUE,**

**+ print.Q=TRUE,**

**+ print.pval.Q=TRUE,**

**+ print.I2=TRUE,**

**+ digits=2)**
