# Supplementary material for: The Efficacy and Safety of Carbon Ion Radiotherapy for Meningiomas: A Systematic Review and Meta-Analysis
Source: Front Oncol. 2021 May 25;11:620534. doi: 10.3389/fonc.2021.620534 (PMC8185343; doi:10.3389/fonc.2021.620534)
Supplement: Supplementary file 2 [file Table_2.docx]

**Supplementary Material**

Search Strategies

| **1. Cochrane Central Register of Controlled Trials (CENTRAL) (12) 2021/3/17 /18:23** |
| --- |
| #1 MeSH descriptor: [Heavy Ions] explode all trees 1 |
| #2 MeSH descriptor: [Heavy Ion Radiotherapy] explode all trees 48 |
| #3 MeSH descriptor: [Protons] explode all trees 105 |
| #4 MeSH descriptor: [Photons] explode all trees 51 |
| #5 "ion" OR "proton" OR "photons" 12143 |
| #6 #1 OR #2 OR #3 OR #4 OR #5 12178 |
| #7 MeSH descriptor: [Meningioma] explode all trees 67 |
| #8 meningeoma OR Meningiomas OR meningoma OR meningothelioma |
| #9 #7 OR #8 172 |
| #10 #6 AND #9 12 |
| **2.Pubmed （362）2021/3/17/18:43** |
| #1 Search: "Heavy Ions"[Mesh] OR "Heavy Ion Radiotherapy"[Mesh] Sort by: Most Recent 5,885 |
| #2 Search: "Photons"[Mesh] Sort by: Most Recent 12,742 |
| #3 Search: "Protons"[Mesh] Sort by: Most Recent 29,527 |
| #4 Search: "Ions"[Mesh] Sort by: Most Recent 730,123 |
| #5 Search: "Meningioma"[Mesh] Sort by: Most Recent 19,595 |
| #6 Search: meningeoma[Title/Abstract] OR meningothelioma[Title/Abstract] OR meningoma[Title/Abstract] OR Meningioma[Title/Abstract] Sort by: Most Recent 16,488 |
| #7 Search: (((("Ions"[Mesh]) OR ("Protons"[Mesh])) OR ("Photons"[Mesh])) OR ("Heavy Ions"[Mesh] OR "Heavy Ion Radiotherapy"[Mesh])) OR ("heavy ion"[Title/Abstract] OR "carbon ion"[Title/Abstract] OR "ion"[Title/Abstract] OR "proton"[Title/Abstract] OR "photons"[Title/Abstract]) Sort by: Most Recent |
| #8 Search: ("Meningioma"[Mesh]) OR (meningeoma[Title/Abstract] OR meningothelioma[Title/Abstract] OR meningoma[Title/Abstract] OR Meningioma[Title/Abstract]) Sort by: Most Recent 1,101,078 |
| #9 Search: (("Meningioma"[Mesh]) OR (meningeoma[Title/Abstract] OR meningothelioma[Title/Abstract] OR meningoma[Title/Abstract] OR Meningioma[Title/Abstract])) AND ((((("Ions"[Mesh]) OR ("Protons"[Mesh])) OR ("Photons"[Mesh])) OR ("Heavy Ions"[Mesh] OR "Heavy Ion Radiotherapy"[Mesh])) OR ("heavy ion"[Title/Abstract] OR "carbon ion"[Title/Abstract] OR "ion"[Title/Abstract] OR "proton"[Title/Abstract] OR "photons"[Title/Abstract])) Sort by: Most Recent 362 |
| **3.EMBASE (12) 2021/3/17/20:25** |
| #1 "Heavy Ions"/exp OR "Heavy Ion Radiotherapy"/exp |
| #2 "Photons"/exp 12,742 |
| #3 "Protons"/exp 29,527 |
| #4 "Ions"/exp 730,123 |
| #5 "Meningioma"/exp 19,595 |
| #6 #1 OR #2 OR #3 OR #4 |
| #7 #5 AND #6 |
| **4.Chinese Biomedical Literature Database (0) 2021/3/17/20:52** |
| #1 "脑膜瘤(主题)"[不加权:扩展] |
| #2 "碳离子(主题)"[不加权:扩展] OR "光子(主题)"[不加权:扩展] OR "质子(主题)"[不加权:扩展] |
| #1 AND #2 |
