# Supplementary material for: The Efficacy and Safety of Carbon Ion Radiotherapy for Meningiomas: A Systematic Review and Meta-Analysis
Source: Front Oncol. 2021 May 25;11:620534. doi: 10.3389/fonc.2021.620534 (PMC8185343; doi:10.3389/fonc.2021.620534)
Supplement: Supplementary file 3 [file Table_3.docx]

e-Table 1 Case series study quality evaluation

| Included Study | A1 | A2 | A3 | A4 | A5 | A6 | A7 | A8 | A9 | A10 | A11 | A12 | A13 | A14 | A15 | A16 | A17 | A18 | A19 | A20 | Number of Yes |
| --- | --- | --- | --- | --- | --- | --- | --- | --- | --- | --- | --- | --- | --- | --- | --- | --- | --- | --- | --- | --- | --- |
| Gudjonsson1999 | Yes | Yes | NO | NO | Unclear | Yes | Yes | NO | NO | Yes | Yes | Yes | Yes | Yes | Yes | Yes | Yes | Yes | NO | Unclear | 13 |
| Hug2000 | Yes | Yes | NO | Yes | Unclear | Yes | Yes | NO | NO | Yes | Yes | Yes | Yes | Yes | Yes | Yes | Yes | Yes | NO | Unclear | 14 |
| Vernimmen2001 | Yes | Yes | NO | NO | Unclear | Yes | Yes | NO | NO | Yes | Yes | Yes | Yes | Yes | Yes | Yes | Yes | Yes | NO | Unclear | 13 |
| Weber2004 | Yes | Yes | NO | Yes | Unclear | Yes | Yes | NO | NO | Yes | Yes | Yes | Yes | Yes | Yes | Yes | Yes | Yes | NO | Unclear | 14 |
| Boskos2009 | Yes | Yes | NO | Yes | Yes | NO | Yes | NO | NO | Yes | Yes | Yes | Yes | Yes | Yes | Yes | Yes | Yes | Yes | Unclear | 15 |
| Comb2010 | Yes | Yes | NO | NO | Yes | Yes | Yes | NO | NO | Yes | Yes | Yes | Yes | Yes | Yes | Yes | Yes | Yes | NO | Unclear | 17 |
| Halasz2011 | Yes | Yes | NO | NO | Yes | NO | Yes | NO | NO | Yes | Yes | Yes | Yes | Yes | Yes | Yes | Yes | Yes | NO | Unclear | 14 |
| Adeberg2012 | Yes | Yes | NO | NO | Yes | NO | Yes | NO | NO | Yes | Yes | Yes | Yes | Yes | Yes | Yes | Yes | Yes | Yes | Unclear | 18 |
| Rieken12012 | Yes | Yes | Yes | NO | Unclear | Yes | Yes | NO | Yes | Yes | Yes | Yes | Yes | Yes | Yes | Yes | Yes | Yes | NO | Unclear | 17 |
| Slater2012 | Yes | Yes | NO | Yes | Yes | NO | Yes | Yes | NO | Yes | Yes | Yes | Yes | Yes | Yes | Yes | Yes | Yes | NO | Unclear | 14 |
| Weber2012 | Yes | Yes | Yes | NO | Unclear | Yes | Yes | NO | Yes | Yes | Yes | Yes | Yes | Yes | Yes | Yes | Yes | Yes | NO | Unclear | 15 |
| Comb2013a | Yes | Yes | Yes | NO | Unclear | Yes | Yes | Yes | NO | Yes | Yes | Yes | Yes | Yes | Yes | Yes | Yes | Yes | NO | Unclear | 17 |
| Comb2013b | Yes | Yes | NO | Yes | Yes | NO | Yes | Unclear | NO | Yes | Yes | Yes | Yes | Yes | Yes | Yes | Yes | Yes | Yes | Unclear | 17 |
| Murray2017 | Yes | Yes | NO | Yes | Yes | NO | Yes | Yes | NO | Yes | Yes | Yes | Yes | Yes | Yes | Yes | Yes | Yes | NO | Unclear | 15 |
| Sanford2017 | Yes | Yes | NO | Yes | Yes | NO | Yes | Yes | NO | Yes | Yes | Yes | Yes | Yes | Yes | Yes | Yes | Yes | NO | Unclear | 17 |
| Vlachogiannis2017 | Yes | Yes | NO | Yes | Yes | NO | Yes | Yes | NO | Yes | Yes | Yes | Yes | Yes | Yes | Yes | Yes | Yes | NO | Unclear | 14 |
| El Shafie2018a | Yes | Yes | NO | Yes | Unclear | NO | Yes | Yes | Yes | Yes | Yes | Yes | Yes | Yes | Yes | Yes | Yes | Yes | NO | Unclear | 16 |
| El Shafie2018b | Yes | Yes | NO | Yes | Unclear | NO | Yes | Yes | Yes | Yes | Yes | Yes | Yes | Yes | Yes | Yes | Yes | Yes | NO | Unclear | 15 |
| Number of Yes | 18 | 18 | 3 | 10 | 8 | 9 | 18 | 7 | 4 | 18 | 18 | 18 | 18 | 18 | 18 | 18 | 18 | 18 | 3 | 0 |  |

A1:Whether the hypothesis, purpose and goal of the study are clearly stated; A2:Whether the characteristics of the patient are described; A3:Whether to collect cases in multiple centers; A4:Whether the inclusion and exclusion criteria are clear and reasonable; A5:Whether the inclusion of patients is continuous; A6:Whether the patient's condition is consistent A7:Whether the main interventions are clearly described; A8:Whether the joint intervention measures are clearly described; A9:Whether to determine the outcome of the study to be measured in advance; A10:Whether to use reasonable objective and / or subjective methods to measure the relevant outcome indicators; A11:Whether the outcome index was measured before and after the intervention; A12:Whether reasonable statistical tests are used to evaluate the relevant outcome indicators; A13:Whether the follow-up time was reported; A14,Whether the loss of follow-up has been reported; A15:Whether the estimation of random variables is provided in the data analysis of the relevant outcome indicators; A16:Whether adverse events related to intervention have been reported; A17:Whether the results of the study support its conclusion; A18:Does it indicate the conflict of interest and the source of support for the research; A19:Whether this study is a prospective study;A20:Whether or not to blind the outcome evaluator
